# Distinct Single Cell Gene Expression in Peripheral Blood Monocytes Correlates With Tumor Necrosis Factor Inhibitor Treatment Response Groups Defined by Type I Interferon in Rheumatoid Arthritis

**DOI:** 10.3389/fimmu.2020.01384

**Published:** 2020-07-16

**Authors:** Theresa L. Wampler Muskardin, Wei Fan, Zhongbo Jin, Mark A. Jensen, Jessica M. Dorschner, Yogita Ghodke-Puranik, Betty Dicke, Danielle Vsetecka, Kerry Wright, Thomas Mason, Scott Persellin, Clement J. Michet, John M. Davis, Eric Matteson, Timothy B. Niewold

**Affiliations:** ^1^Department of Medicine, Colton Center for Autoimmunity, New York University School of Medicine, New York, NY, United States; ^2^Division of Rheumatology, Department of Medicine, New York University School of Medicine, New York, NY, United States; ^3^Department of Rheumatology, Ren Ji Hospital, Shanghai Jiao Tong University School of Medicine, Shanghai, China; ^4^Department of Pathology, Immunology and Laboratory Medicine, University of Florida College of Medicine, Gainesville, FL, United States; ^5^Division of Rheumatology, Department of Internal Medicine, Mayo Clinic, Rochester, MN, United States

**Keywords:** type I interferon, tumor necrosis factor-alpha, monocyte, single cell, rheumatoid arthritis, janus kinase 1, interferon-beta, interferon-alpha

## Abstract

Previously, we demonstrated in test and validation cohorts that type I IFN (T1IFN) activity can predict non-response to tumor necrosis factor inhibitors (TNFi) in rheumatoid arthritis (RA). In this study, we examine the biology of non-classical and classical monocytes from RA patients defined by their pre-biologic treatment T1IFN activity. We compared single cell gene expression in purified classical (CL, *n* = 342) and non-classical (NC, *n* = 359) monocytes. In our previous work, RA patients who had either high IFNβ/α activity (>1.3) or undetectable T1IFN were likely to have EULAR non-response to TNFi. In this study comparisons were made among patients grouped according to their pre-biologic treatment T1IFN activity as clinically relevant: “T1IFN undetectable (T1IFN ND) or IFNβ/α >1.3” (*n* = 9) and “T1IFN detectable but IFNβ/α ≤ 1.3” (*n* = 6). In addition, comparisons were made among patients grouped according to their T1IFN activity itself: “T1IFN ND,” “T1IFN detected and IFNβ/α ≤ 1.3,” and “IFNβ/α >1.3.” Major differences in gene expression were apparent in principal component and unsupervised cluster analyses. CL monocytes from the T1IFN ND or IFNβ/α >1.3 group were unlikely to express *JAK1* and *IFI27* (*p* < 0.0001 and *p* 0.0005, respectively). In NC monocytes from the same group, expression of *IFNAR1, IRF1, TNFA, TLR4* (*p* ≤ 0.0001 for each) and others was enriched. Interestingly, *JAK1* expression was absent in CL and NC monocytes from nine patients. This pattern most strongly associated with the IFNβ/α>1.3 group. Differences in gene expression in monocytes among the groups suggest differential IFN pathway activation in RA patients who are either likely to respond or to have no response to TNFi. Additional transcripts enriched in NC cells of those in the T1IFN ND and IFNβ/α >1.3 groups included MYD88, CD86, IRF1, and IL8. This work could suggest key pathways active in biologically defined groups of patients, and potential therapeutic strategies for those patients unlikely to respond to TNFi.

## Introduction

Rheumatoid Arthritis (RA) is the most common inflammatory joint disease world-wide, characterized by a destructive arthritis and serious extra-articular manifestations, including accelerated vascular disease (1). Early, effective treatment prevents damage. Thus, remission or very low disease activity within the first 3 months is the goal (2, 3). New therapies have made remission possible for a greater number of patients. However, the current treatment strategy is one of trial-and-error, as we are not able to predict which medication will work for an individual patient. Tumor necrosis factor inhibitors (TNFi) are the most common biologic treatment employed (4). Responses are variable, with ~30% not responding and another 30% achieving only partial response. Insufficient treatment is associated with increased morbidity, mortality, and a heavy economic burden (5–8). Type I IFN (T1IFN) levels are genetically determined to some degree (9, 10) and T1IFNs are pleiotropic biologic response modifiers, making them ideal candidate biomarkers for response to immunomodulatory therapies. Recently, we studied pre-treatment circulating IFN-alpha (IFNα), IFN-beta (IFNβ), and total T1IFN activity in RA patients just prior to receiving a TNFi (11) in independent test and validation cohorts. The ratio of IFNβ to IFNα activity (IFNβ/α) > 1.3 was strongly predictive of non-response to TNFi therapy (specificity = 77% in the validation cohort). Remarkably, no patient with a ratio >1.3 achieved remission or low disease activity.

Monocytes are one of the major effector cells in RA (12, 13). Classical (CD14++CD16-) and non-classical (CD14dim, CD16+) monocytes have been implicated in pathogenesis (14–17) and, the frequency of intermediate (CD14++CD16+) monocytes has been found to associate with disease severity (18). In general, intermediate and non-classical monocytes emerge sequentially from classical monocytes (19). By studying classical (CL) and non-classical (NC) monocytes from RA patients defined by their pre-biologic treatment type I IFN activity, we can understand the functional impact of the IFN ratio on a key effector cell type. In addition, we can identify other differences in major immunologic pathways that may lend understanding to TNFi response in such patients.

We chose to study gene expression in single cells, as effects of IFN on single immune cells or cell types may be masked in whole blood or mixed cell populations (20). NC monocytes are a small percentage of circulating monocytes, and have been less frequently studied in humans. By purifying the monocyte subsets prior to single cell isolation, we were able to intentionally increase our number of NC monocytes to study. Measurement of expression of genes by qPCR [rather than by more broad-based methods such as droplet RNA-seq (21)] allowed us to deeply examine gene expression in a rare cell population. We find that circulating T1IFN ratio corresponds to strikingly different gene expression patterns in the RA patient monocytes, and that particular transcripts such as *JAK1* are highly informative and could suggest alternate therapeutic avenues in patients who are predicted to be TNFi non-responders.

## Materials and Methods

### Patient and Public Involvement

Patients/the public were not involved in the design of the study. The study design and plans to disseminate study results to participants were informed by patient priorities and preferences.

### Patients and Samples

Blood samples from 15 patients with RA were recruited from the Mayo Clinic in Rochester, Minnesota, USA. All of the patients fulfilled the 2010 American College of Rheumatology classification criteria for RA (22) and were seropositive. Exclusion criteria included overlap autoimmune connective tissue disease, pregnancy, active acute infection, chronic infection (e.g., hepatitis C, HIV, etc.), current intravenous therapy (e.g., methylprednisolone or cyclophosphamide), and history of biologic therapy. All samples were obtained prior to initiation of biologic therapy and all patients were naïve to biologic and to kinase inhibitor therapy. All patients provided informed consent, and the study was approved by the institutional review board.

In our previous test and validation cohort study, patients with undetectable T1IFN activity typically did not respond to TNFi therapy (11). Thus, to examine the biology of monocytes from groups of patients according to their likely TNFi response, these patients were grouped together with those who have an IFNβ/α ratio > 1.3 [those likely to have non-response, (11)]. For initial analysis, subjects were grouped by their pre-biologic treatment serum T1IFN activity into two groups, those with detectable T1IFN activity but low IFNβ/α ratio (IFNβ/α > 0 and ≤ 1.3, *n* = 6), and those with either undetectable T1IFN activity or a high IFNβ/α ratio (T1IFN ND or >1.3, *n* = 9). To examine the possible influence of the IFNβ/α activity on the cells, (11) we also compared gene expression among three groups: those with undetectable T1IFN activity (T1IFN ND, *n* = 3), those with detectable T1IFN activity but low IFNβ/α ratio (IFNβ/α ≤ 1.3, *n* = 6), and those with a high IFNβ/α ratio (IFNβ/α >1.3, *n* = 6).

### Determination of IFNβ/α Ratio

T1IFN activity in serum was measured using a validated functional assay in which reporter cells are used to measure the ability of patient sera to cause T1IFN-induced gene expression (23). Reporter cells (WISH cells, ATCC #CCL-25) were cultured with patient serum for 6 h. Cells were then lysed, and cDNA was made from total cellular mRNA. Canonical T1IFN-induced gene expression (*MX1, PKR*, and *IFIT1*) (24), was measured using qPCR. The relative expression of these three genes was standardized to healthy donors and summed to generate a score reflecting the ability of sera to cause T1IFN-induced gene expression (serum T1IFN activity). This assay has been informative in a wide range of autoimmune diseases (11, 23, 25–27), and we have not found significant functional inhibitors in samples studied to date (28). Additional aliquots were tested following pre-incubation with polyclonal anti-IFNα (19.6 μg/mL, PBL Assay Science) and anti-IFNβ (10.1 μg/mL, Chemicon) antibodies. The amount of inhibition of the observed T1IFN activity by anti-IFNα antibody allowed for quantitative assessment of IFNβ activity, and that by antiβ antibody allowed for quantitative assessment of IFNα activity. The ratio of IFNβ activity to IFNα activity (IFNβ/α activity ratio) was then calculated for each serum sample using these data. Those samples reading very low (<1 pg/mL) for total T1IFN activity were categorized as not having significant T1IFN present, and no ratio was calculated.

### Purification of Classical (CD14^++^CD16^−^) and Non-classical (CD14^dim^CD16^+^) Monocytes

Classical (CL) and non-classical (NC) monocytes were isolated from peripheral blood using the protocol described in Jin et al. (29) (Supplemental Figure 1). Briefly, CL (CD14++CD16–) monocytes were purified using the Human Pan-Monocyte Isolation Kit (Miltenyi) with modification of adding anti-CD16-biotin (Miltenyi) into the biotin–antibody cocktail. CD14+ selection (Miltenyi) was used subsequently to further increase purity. Purified CL monocytes were stained with Molecular Probes CellTracker Green CMFDA Dye (Life Technologies). NC (CD14^dim^CD16+) monocytes were purified similarly, using CD16 microbeads (Miltenyi) during positive selection. Purity checked by flow cytometry was very high (>95%) for both CL and NC monocytes (Supplemental Figure 1).

### C1 Single Cell Isolation and Measurement of Gene Expression

Using the Fluidigm C1 Single-Cell Auto Prep System we isolated single cells from the bulk monocyte subsets. NC and CL monocytes were loaded onto the C1 Integrated Fluidic Circuit (IFC) sequentially. Determination of NC or CL lineage of individual cells was made by direct visualization using fluorescent microscopy. Pre-amplification was done using the C1 Single-Cell Auto Prep Array IFC, according to the manufacturer's protocol. Melt curves were inspected to ensure that all PCR products were uniform. Amplification curves were analyzed and those not following the expected log-growth curve were excluded. Target genes included major cytokines and pathway proteins involved in inflammation (Supplemental Table 1). Target gene pre-amplified cDNAs were assayed using 96.96 IFCs on the BioMark HD System (Fluidigm) according to the manufacturer's protocol. Empty wells and wells that contained more than one cell after C1 automated single cell capture were identified by visual inspection using microscopy. These wells were excluded from the dataset. A cell was also removed from the dataset if failure score (total CT value) was >2 standard deviations above the mean, as this indicated that the cell's overall expression level was too low to be trusted for downstream analysis.

### Statistical Analysis

Principal component analysis was used to reduce dimensionality in the complex data sets, and compare overall trends between patient groups and CL and NC monocytes. Unsupervised hierarchical clustering was done to detect individual genes and gene sets that defined the patient groups and to identify other possible strata within the data. Each gene was also tested individually for association with patient group, using Mann Whitney *U*-testing for the quantitative data and Fisher's exact test for the categorical expressed/not expressed analysis. For these analyses, we used the following strategy to account for multiple comparisons. We expected to find correlations between transcripts in the same cell, which would make a strict Bonferroni correction inappropriate, as each of the 87 tests is not independent. We first calculated pairwise Spearman correlations (rho) for each possible pairing of transcripts, resulting in 3,741 pairwise correlations. The average correlation between transcripts was then calculated and a threshold *p*-value was derived using the following modified Bonferroni method to account for between-transcript correlations:

$$\begin{array}{l} p_{corr\;}=p_{obs}\;x\;87\left[\frac{1-\;\sum_{i=1}^{3741}|rho_{i}|}{3741}\right] \end{array}$$

This resulted in a threshold *p*-value of <0.0008 for a corrected alpha of 0.05.

## Results

### Circulating Type I IFN Ratio Corresponds to Large Differences in Monocyte Gene Expression

**Figure 1:**
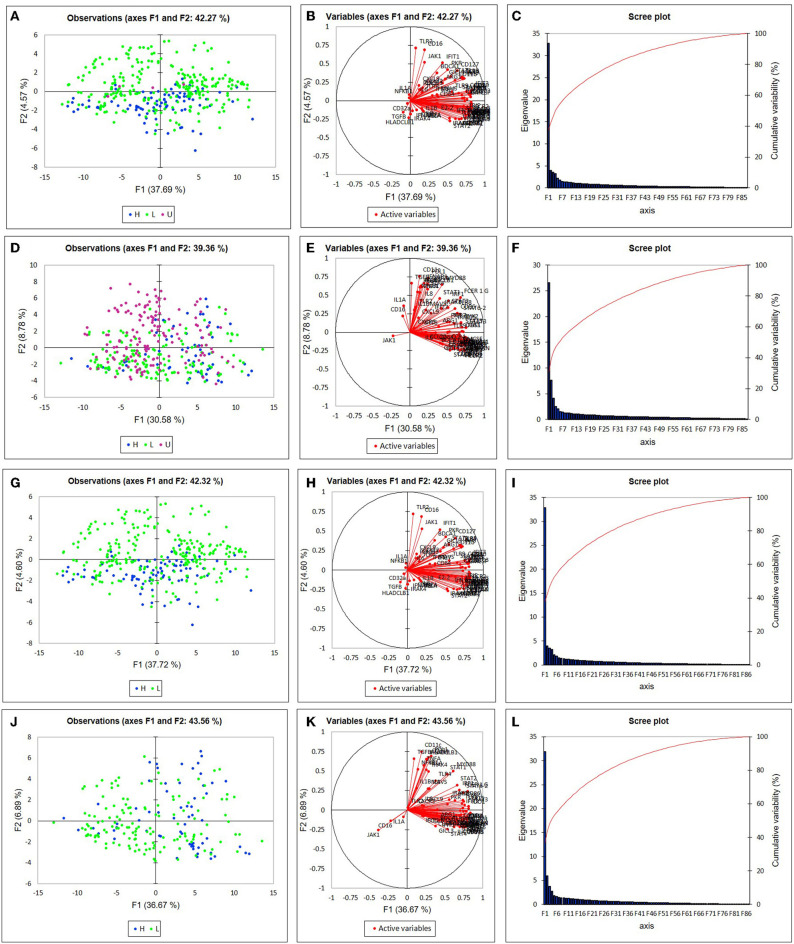
Principal component analysis (PCA) of single monocyte gene expression. Plots depict the first two differentiating factors among the gene expression data of non-classical monocytes **(A,G)**, and classical monocytes **(D,J)**. Blue (•) denotes data from subjects in the “T1IFN ND or > 1.3” group (*n* = 92 for CL, *n* = 63 for NC). Green (•) denotes data from subjects in the “IFNβ/α ≤ 1.3” group (*n* = 246 for CL, *n* = 142 for NC). Purple (•) denotes data from subjects in the “T1IFN undetected” group (*n* = 4 for CL, *n* = 154 for NC). In **(G–L)** T1IFN undetected cells were excluded from analysis. In **(A,G)**, most of the monocytes from patients in the IFNβ/α > 1.3 group are on one side of the Y axis. In **(D,J)**, most of the monocytes from patients in the IFNβ/α > 1.3 group are on one side of the X axis. Thus, the pretreatment T1IFN-β/α ratio appears to impact monocyte gene expression in subjects in the IFNβ/α > 1.3 group. Active variables **(B,E,H,K)** and scree plots **(C,F,I,L)** for monocyte PCA are shown.

**Figure 2:**
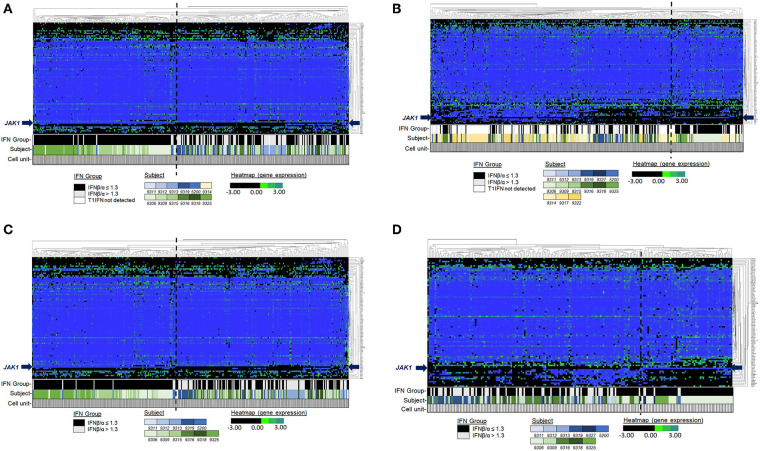
Unsupervised hierarchical clustering of single cell pre-biologic gene expression in monoctyes. Genes (Y-axis). Single monocytes (X-axis). Both genes and cells were selected for clustering. The bars under the heatmap indicate the T1IFN group, subject, and width of data depicted that is from a single cell. Each subject is depicted by a different color: blue shading indicates the cell is from a subject in the IFNβ/α > 1.3 group; green shading indicates the cell is from a subject in the IFNβ/α ≤ 1.3 group; gold shading indicates the cell is from a subject in the T1IFN undetected group. In **(A,B)**, T1IFN undetected cells were included in the analysis. In **(C,D)**, cells from the T1IFN undetected group were excluded from analysis. See legends for relative expression level (black/green/blue) and T1IFN group (black/gray/white) color assignments.

Among the participants in the groups, there were no significant differences in age or disease activity score (DAS), and treatments were comparable (Table 1). We were able to analyze results of 87 target genes from 701 individual monocytes (342 CL, 359 NC) (Supplemental Tables 2, 3). Principal component analysis (PCA) revealed apparent differences between high and low IFNβ/α groups in the first component in NC Mo and in the second component in CL Mo when cells were labeled by their T1IFN group category (Figure 1). The first component's influence was not as strong in NC cells when cells from patients with undetectable T1IFN activity were included (F1 30.58%, Figure 1D) in comparison to when they were not included (F1 36.67%, Figure 1J), supporting that the T1IFN activity influences NC Mo gene expression in a way that contributes to the pattern explaining the greatest difference detected between the two groups. We next performed unsupervised hierarchical clustering of the target genes to visualize the difference between groups with respect to individual transcripts (Figure 2). In this analysis, it was clear that certain genes strongly aligned with the T1IFN activity groups. In particular, *JAK1* appeared to be strongly predictive of patient group (Figure 2). The association was strongest when the clustering is done without including the cells from patients who did not have detectable T1IFN activity (Figures 2C,D).

**Table 1:** General characteristics, disease activity measures, and medications of RA patients.

**Characteristic**	**T1IFN ND *or* IFNβ/α > 1.3 (*n* = 9)**	**T1IFN detected but IFNβ/α ≤ 1.3 (*n* = 6)**	***P*-value (*T*-test)**
Age (mean, range)*	54 (27–75)	55 (42–70)	0.87
Gender (F, M)	4F, 5M	4F, 2M	-
CCP positivity (*n*, %)	8 (89)	6 (100)	-
RF positivity (*n*, %)	7 (78)	5 (83)	-
ESR (median, range)	7.5 (0–41)	21.5 (13–40)	0.28
DAS28-CRP (mean ± std.dev, range)*	2.92 ± 1.82	3.42 ± 0.62	0.57
	1.15–5.77	2.52–4.23	-
**Medications (*****n*****, %)**			
Prednisone	4 (44)	1 (17)	-
*median dose (mg/day)*	*10*	*5*	-
*dose range (mg/day)*	*5 - 15*	*5*	-
NSAIDs (prn)	0	3 (50)	-
Methotrexate	8 (89)	3 (50)	-
*median dose (mg/wk)*	*20*	*10*	0.28
*dose range (mg/wk)*	*17.5 - 25*	*7.5 - 20*	-
Sulfasalazine	2 (22)	0	-
Leflunomide	2 (22)	0	-
Hydroxychloroquine	5 (55)	2 (33)	-
Statin	3 (33)	1 (17)	-
ASA-81	2 (22)	0	-
Allopurinol	1 (11)	0	-

### Different Genes Were Associated With Blood IFN Ratio in CL vs. NC Cells

In the categorical expressed/not expressed analysis, there were significant differences in the transcripts observed between groups in CL as compared to NC monocytes. In CL monocytes, in addition to JAK1, IFI27 (an interferon-stimulated gene [ISG]) was less likely to be expressed in the T1IFN ND or IFNβ/α > 1.3 group (Table 2). In NC monocytes, a number of transcripts were more likely to be expressed in the T1IFN ND or IFNβ/α > 1.3 group. These include HLADRB1, TNFA, PDL1, TGFB, CD11c, IL8, and IFNAR1 (Table 2). One transcript, JAK1, was less likely to be expressed in the T1IFN ND or IFNβ/α > 1.3 group (Table 2).

**Table 2:** Odds of being expressed in the TNFi non-response (T1IFN ND or > 1.3) group.

**Mo**	**Transcript**	**Odds ratio**	**95% CI**	***P*-value**
CL	*JAK1*	0.061	0.030–0.126	<0.0001
CL	*IFI27*	0.380	0.229–0.637	0.0005
NC	*HLADB1*	3.23	1.953–5.198	<0.0001
NC	*TNFA*	2.96	1.780 to 4.985	<0.0001
NC	*PDL1*	2.64	1.703–4.155	<0.0001
NC	*TGFB*	2.61	1.674–4.126	<0.0001
NC	*CD11c*	2.58	1.631–4.002	<0.0001
NC	*IL8*	2.46	1.595–3.779	<0.0001
NC	*JAK1*	0.39	0.248–0.617	<0.0001
NC	*IFNAR1*	2.14	1.382–3.304	0.0008

*P-value by Fisher's Exact test. Mo, monocyte; CL, Classical; NC, Non-classical*.

Examination of the quantitative data between T1IFN ND or IFNβ/α > 1.3 and T1IFN (T1IFN) detected but IFNβ/α ≤ 1.3 groups resulted in some similar findings and some additional findings became apparent. In CL monocytes, many genes were reduced in expression in the T1IFN ND or IFNβ/α > 1.3 group, including JAK1 and IFI27 which were significant in the expressed/not expressed analysis. Additional transcripts that were reduced in the T1IFN ND or > 1.3 group in quantitative analysis were: TLR7, TLR8, TLR2, MAVS, PKR, GMCSF, IRF8, IL4, IL1A, ILT7, CD127, CD16, and CCR4 (Supplemental Figure 2). In NC Mo, a large number of genes showed increased expression in the T1IFN ND or IFNβ/α > 1.3 group, including HLADRB1, TNFA, PDL1, TGFB, CD11c, IL8, and IFNAR1 which were identified in the expressed/not expressed analysis. Additional genes that were significantly increased in the in the T1IFN ND or > 1.3 group in quantitative analysis were: TLR4, MYD88, IRF1, FCER1G, and CD86 (Supplemental Figure 3). Genes that differed by non-parametric (Mann Whitney *U*) univariate analysis between the clinically relevant groups (T1IFN ND or IFNβ/α > 1.3 and T1IFN detected but ≤ 1.3) were tested in multivariate logistic regression models. In CL Mo, JAK1, TLR2, IRF8, CD16, and IL1A were retained as independent factors predictive of patient group (Supplemental Table 4). In NC Mo, CD86, HLADRB1, IL8, PDL1, TGFB, and FCER1G were retained in the model (Supplemental Table 4). ROC curve analysis of these transcripts demonstrated an area under the curve (AUC) of 0.89 (Std. error 0.0172, 95% CI 0.849–0.919) and 0.76 (Std. error 0.026, 95% CI 0.708–0.799), respectively.

Examination of the quantitative data between the three T1IFN activity groups (T1IFN ND, IFNβ/α > 1.3 and IFNβ/α ≤ 1.3) allowed us to detect differences in monocyte gene expression that may be more directly associated with T1IFN activity than with EULAR TNFi treatment response. There were too few CL monocytes in the T1IFN ND group to allow for comparison, and comparison between the IFNβ/α > 1.3 and IFNβ/α ≤ 1.3 groups with the cells from the T1IFN ND patients omitted resulted in similar findings as when the T1IFN ND cells were included with the IFNβ/α > 1.3 group (Supplemental Figure 4). However, the number of NC monocytes in the T1IFN ND group was sufficient (*n* 154). The following transcripts were more highly expressed in NC monocytes in both the T1IFN ND and the IFNβ/α > 1.3 group in comparison to the IFNβ/α ≤ 1.3 group: MYD88, IRF1, IL8, and CD86 (Supplemental Figure 5). Expression of following transcripts did not differ in NC monocytes between T1IFN ND and IFNβ/α ≤ 1.3 groups, but were more highly expressed in the IFNβ/α > 1.3 group: TRAF6, PRDM1, IL2, IL5, IL12, TLR3, TLR7, IFIH1, IFIT2, CTLA4, CCR2, and CCR5 (Supplemental Figure 5).

### JAK1 Expression Was Completely Suppressed in Some RA Patients, and This Suppression Correlated Strongly With Type I IFN Activity Previously Found to Predict Non-response (T1IFN ND or > 1.3)

**Figure 3:**
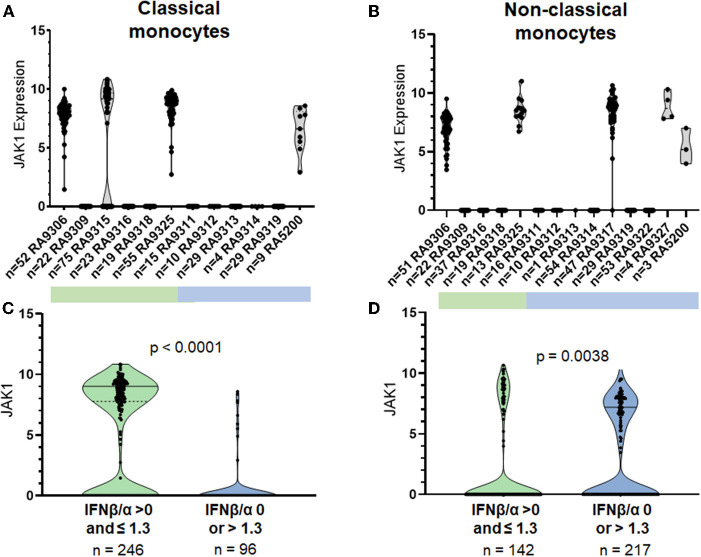
Expression of JAK1 in single classical and non-classical monocytes. *P*-value by non-parametric Mann Whitney *U*. Top panels **(A,B)** show each individual patient's cells in a separate column, bottom panels **(C,D)** show cells from all patients in aggregate.

*JAK1* was unlikely to be expressed in both CL and NC monocytes from patients in the T1IFN ND or > 1.3 group (Odds 0.06, *p*-value <0.0001, 95% CI 0.03–0.13 in CL; Odds 0.39, corrected *p*-value <0.0063, 95% CI 0.25–0.62 in NC). Ninety-one percent of CL monocytes and 76% of NC monocytes in the T1IFN ND or > 1.3 group did not express JAK1. Whereas, in the other IFN group (T1IFN detected but IFNβ/α ≤ 1.3), the majority of CL monocytes (63%) and 45% of NC monocytes expressed JAK1. Strikingly, only one participant in the T1IFN ND or IFNβ/α > 1.3 group expressed JAK1 in CL monoctyes (Figure 3). Interestingly, after enrollment into our study, this participant was found to have several pre-malignant melanoma lesions that were ultimately removed near the time she began TNFi. Melanocytes and melanoma cells produce IFNβ and are capable of suppressing their own proliferation by secretion of endogenous IFNβ (30). It is possible that the increased IFNβ/α activity noted in this participant was significantly influenced by the pre-malignant melanoma and less informative as a physiologic immune phenotype previously found to be predictive of response to TNFi.

The participants who did not express JAK1 in CL monocytes also did not express JAK1 in NC monocytes (Figure 3, Supplemental Figure 6). A difference in NC expression of JAK1 between groups was present (*p* < 0.0001) when cells from patients in the T1IFN ND group were omitted (Supplemental Figure 6). In the JAK1 expressers, most of the cells expressed JAK1 (83% of cells in CL, 99% in NC). The “on or off” expression pattern was not seen in any of the other transcripts studied and was not observed in healthy controls (Supplemental Figure 7). Given the observed distribution, even a subject with only 5 cells represented would have an extremely small (0.01%) chance for miscategorization due to sampling error (29). Each of the 96-well plates were run independently on different days, and it would be highly unlikely that the same one out of the 87 target genes would fail each time, and largely in those patients with a high IFN activity ratio (IFNβ/α > 1.3). The same primers were used to measure expression in all experiments, and also have been used to study healthy controls (Supplemental Figure 7) and lupus patients in other studies [Supplemental Figure 8 and (29)]. Interestingly, one lupus patient studied demonstrated the “on/off” pattern (Supplemental Figure 8). In addition, the “on/off” pattern was observed in other transcripts of major pathway proteins in lupus patients and not in healthy controls (example shown in Supplemental Figure 8).

## Discussion

Using a novel single cell PCR approach, we used a panel of T1IFN and innate immune system related genes to define gene expression states in monocytes from RA patients grouped by their pre-treatment blood T1IFN activity. Patients were grouped by serum IFNβ/α activity. This is based on our previous study, which showed in test and validation cohorts that patients with IFNβ/α activity > 1.3 were not likely to respond to TNFi therapy, and that those with undetectable T1IFN were also not likely to respond. Comparisons between these groups allowed us to examine the biology of monocytes in RA patients who would be less likely to respond to a TNFi. Comparisons were also made among the three groups T1IFN activity, which allowed for more direct evaluation of possible impact of the T1IFN activities on monocytes in RA patients.

We observed striking differences in gene expression patterns in circulating CL and NC monocytes between RA patient groups. Our data suggest differential IFN pathway activation in monocyte subsets from RA patients who have elevated IFNβ/α activity (>1.3). The outcome of T1IFN receptor engagement depends on the pathway components present in the cell and the context (e.g., other cytokines can influence the outcome of IFN receptor ligation) (31, 32). Murine data has shown that JAK1 plays an essential and non-redundant role in biologic responses induced by class II cytokine receptor family members, including the receptors for T1IFNs, type II IFN, and IL-10 (33). JAK1 is required for canonical T1IFN pathway signaling. In our data, it was striking that JAK1 expression was absent in some patients' monocytes, and that this was a strong predictor of T1IFN group. The pattern in which none of the monocytes studied in a given subject expressed JAK1 was observed only in patients and not in controls, suggesting that a disease related factor may be contributing to this pattern, such as a cytokine signal inducing a strong transcriptional repressor. It is possible that the T1IFNs could contribute to this process, given that this pattern was associated with IFN ratio in our study.

**Figure 4:**
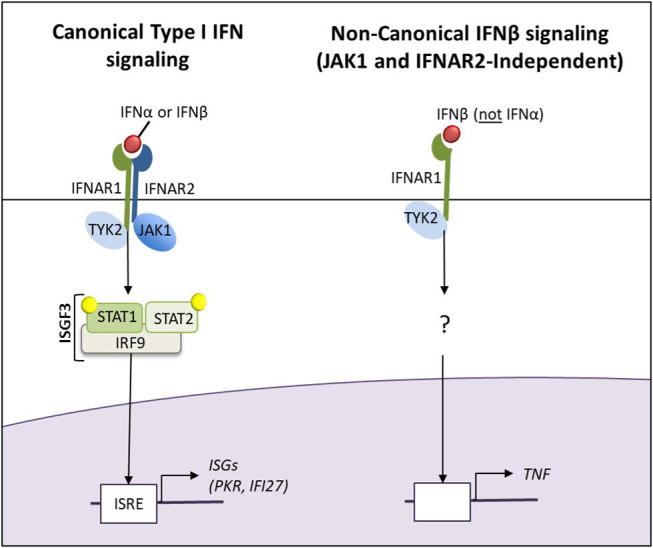
The canonical Type I IFN and non-canonical JAK1-and IFNAR2-independent IFNβ signaling pathways. The pattern of gene expression that differed between the patient groups could suggest that canonical T1IFN pathway signaling is increased in peripheral blood CL monocytes of RA patients who are likely to respond to TNFi, whereas Jak/STAT-independent IFNB-IFNAR1 signaling is increased in NC monocytes of those who are not likely to respond to TNFi.

In this study, (34–38) when patients were grouped by clinical relevance (T1IFN ND or IFNβ/α > 1.3 compared to T1IFN detected but IFNβ/α ≤ 1.3), data suggest differential T1IFN pathway activation between groups. In addition to JAK1, the IFNAR1, IFI27, PKR, and TNFA transcripts were differentially expressed between the two groups. IFNAR1 expression, but not IFNAR2, was enriched in NC Mo of participants in the T1IFN ND or > 1.3 group. IFNAR1 functions in general as a heterodimer with IFNAR2, and canonical signaling through the T1IFN receptor requires JAK1 and results in expression of interferon stimulated genes (ISGs), including IFI27 and PKR. Intriguingly, IFNAR1 can form an active IFNB receptor by itself and activate signaling that does not involve activation of the Jak/STAT pathway (39). Among the transcripts uniquely upregulated by IFNAR1-IFNB signaling was TNFA (39), which in our study, together with IFNAR1, was increased in NC monocytes of participants in the T1IFN ND or IFNβ/α > 1.3 group. JAK1, PKR, and IFI27 were increased in CL monocytes in the T1IFN detectable but IFNβ/α ≤ 1.3 group. The pattern of gene expression that differed between the patient groups could suggest that canonical T1IFN pathway signaling is increased in peripheral blood CL monocytes of RA patients who are likely to respond to TNFi, whereas Jak/STAT-independent IFNB-IFNAR1 signaling is increased in NC monocytes of those who are not likely to respond to TNFi (Figure 4). Recently, Firestein et al. found joint location-specific JAK-STAT signaling in fibroblast-like synoviocytes from RA patients, associated with differential chromatin accessibility of JAK1 and difference in Jak-inhibitor treatment response (40). While we focused on peripheral blood monocytes in this study, we wonder whether similar processes are occurring in the synovial tissue in monocyte-derived cells. In synovium, it is thought that IFNβ can drive subsequent inflammatory cytokine production (41), and it is possible that the differential IFN pathway activation and IFN-priming phenomena we observe in circulating monocytes will relate to macrophages in the inflamed joint.

Our data also suggest that the frequency of osteoclast precursor cells (and consequently, osteoclastogenesis) may be increased in the T1IFN ND or IFNβ/α > 1.3 group. Osteoclastogenesis is controlled by interaction of RANK expressed on osteoclast precursors with its ligand (RANKL) expressed on osteoblasts, synovial fibroblasts, and Th17 cells (42) (Supplemental Figure 10). We found increased expression of TRAF6, which is one of the major gene expression markers of osteoclast precursor cells, in NC monocytes in the high IFNβ/α group compared to both the T1IFN ND and the low IFNβ/α group. Our suspicion that the elevation in TRAF6 in NC monocytes supports increased osteoclastogenesis is in keeping with data from a murine arthritis model (hTNFtg) (16). In hTNFtg arthritis, the numbers of non-classical monocytes in blood are significantly correlated with histological signs of joint destruction and non-classical monocytes display an increased capacity to differentiate into OCs, associated with increased expression of TRAF6 which leads to an increased responsiveness to RANK (16). As illustrated in Supplemental Figure 10, lack of JAK1 expression would also support a likely increase in differentiation to osteoclast precursor cells. Interactions of RANK with RANKL activates pathways that promote production of IFNβ and osteoclastogenesis. IFNβ promotes transcription of ISGs that inhibit osteoclastogenesis (43). The canonical T1IFN pathway is JAK1-dependent. Thus, despite elevated IFNβ, patients in the IFNβ/α > 1.3 group (who are not likely to express JAK1) are less likely able to inhibit osteoclastogenesis.

Expression of CD86 was increased in NC monocytes from both the IFNβ/α > 1.3 and the T1IFN ND groups in comparison to the IFNβ/α ≤ 1.3 group (Supplemental Figure 5). CD80 and CD86 are involved in T cell costimulation and regulation of osteoclastogenesis. CD86 is typically expressed at low levels on monocytes and is upregulated by IFNγ stimulation. Data from murine and human studies by Bozec et al. demonstrated that engagement of CD80/86 by CTLA-4 is an important negative regulatory signal for osteoclast differentiation (44). In their work, targeting CD80/86 by abatacept, a CTLA-4–immunoglobulin fusion protein, reduced, whereas blockade of CTLA-4 by ipilimumab antibody enhanced, the frequency of peripheral osteoclast precursors and osteoclastogenesis (44, 45). Expression of CTLA4 was also increased in NC monocytes in the IFNβ/α > 1.3 group in comparison to the other groups. The role of CTLA4 expression in monocytes has not yet been elucidated. Most is not membrane-bound (45); thus, it is possible that CTLA4 is secreted and acts in a paracrine or autocrine manner to decrease monocyte differentiation into osteoclasts. It will be important to follow-up these findings with additional studies. If increased osteoclastogenesis is confirmed in patients with either no detectable T1IFN or IFNβ/α > 1.3 in comparison to detectable but IFNβ/α ≤ 1.3, abatacept (which is already FDA-approved for treatment of RA) may be a more ideal than a TNFi as initial biologic therapy in this subset of patients.

TRAF6 is also a key protein in inflammatory signaling pathways downstream of MYD88 (Supplemental Figure 11). Expression of MyD88, which is the canonical adaptor downstream of members of the Toll-like receptor (TLR) and interleukin-1 (IL-1) receptor families (46), was increased in both the T1IFN ND and high IFNβ/α groups. Number of osteoclast precursors may be increased, and/or induction of inflammatory cytokines and chemokines through MYD88-dependent TRAF6 pathways may be more active in these patients. As might be expected in patients with increased IFNβ activity, in our study expression of PRDM1 (a negative regulator of IFNβ) was increased in NC monocytes of the IFNβ/α > 1.3 group in comparison to the other two groups. So too were expression of TLR3 and IFIH1 (which encodes MDA5, a cytosolic nucleotide sensor). TLR3 and IFIH1 stimulate IFNβ production (Supplemental Figure 11).

Data regarding IL8 and IRF1 in human monocytes is limited. Potentially relevant IRF1 data comes from knowledge gained regarding myeloid dendritic cells and macrophages. In myeloid dendritic cells, activation of the IFN gamma receptor culminates in transcription of IRF1. Activation of endosomal TLR9 results in activation of IRF1, which translocates to the nucleus and prompts transcription of IFNβ (47). In macrophages, TNF-α induces IFN-β via IRF1 and can induce an IFN-β autocrine loop that acts in synergy with canonical TNF signals to induce sustained expression of inflammatory genes and delayed expression of STAT1-dependent IRGs that prime cells for enhanced responses to subsequent challenge (48). Thus, elevated IRF1 in both the T1IFN ND and IFNβ/α > 1.3 groups may indicate that the IRF1 pathway is more active, and, IFNβ produced may be priming the NC monocytes for amplified responses to stimuli. IL-8 has been found to be increased in RA patient sera and *in vitro* studies have found that IL-8 contributes to resistance of monocyte apoptosis in RA (49). Elevated IL-8 in NC monocytes from patients in both the T1IFN ND and IFNβ/α > 1.3 groups suggests that these patients may have greater resistance to apoptosis in comparison to the T1IFN detectable but IFNβ/α ≤ 1.3 group (49). Resistance to apoptosis may or may not be reduced by TNFi therapy. Thus, follow-up studies that include prospective data are needed.

In summary, in this study we measured gene expression in single monocytes from seropositive RA patients prior to biologic treatment. Our aim was to discover differentiating transcripts among major inflammatory pathways in clinically meaningful groups, which can shed light on possible influences of T1IFN as well as other mediators in RA (e.g., biology of the monocytes themselves in those not likely to respond to TNFi). Thus, we compared data between groups defined by T1IFN activity found to be predictive of TNFi treatment response in our previous work (T1IFN ND or IFNβ/α > 1.3 compared to T1IFN detected but IFNβ/α ≤ 1.3). We also compared data among the T1IFN groups separately (T1IFN ND, IFN detected but IFNβ/α ≤ 1.3, and IFNβ/α > 1.3), which allowed us to more directly interrogate the possible influence of T1IFN itself on the monocytes. We found major differences in monocyte gene expression between the groups, supporting downstream effects upon a critical effector cell population. Interestingly, JAK1 expression was a strong predictor of group, and also was observed to be completely lacking in some patient's monocytes. Our data suggest differences in IFNβ/α activity may skew canonical vs. non-canonical IFN pathway activation in RA patient monocytes. We were able to examine gene expression in NC monocytes among all three T1IFN groups. NC monocytes in patients who, based on our previous work, would be unlikely to respond to TNFi (T1IFN ND and IFNβ/α > 1.3 groups) demonstrated an expression pattern of key transcripts that suggest increased osteoclastogenesis and resistance to apoptosis.

This cross-sectional study does not allow us to determine whether or not the participants responded to biologic therapy. Increased osteoclastogenesis and resistance to apoptosis have been reported in RA patients previously (unrelated to treatment response), and it is interesting that they were different among the RA patient groups in our study. Additional mechanistic studies are needed to determine whether these pathways are indeed influenced by T1IFNs. In our study, ESR and DAS28-CRP did not differ statistically between the groups. However, prospective study in a larger group of patients is needed to determine whether the gene expression pattern that suggests increased osteoclastogenesis and resistance to apoptosis relates to treatment response. Additional limitations of this study include the number of patients studied. As is common with single cell gene expression studies, a large number of cells are studied from a more modest number of people. Despite this, we observed fascinating patterns that were shared across different subjects. The technology we used to capture cells does not capture as many cells as some other platforms, such as droplet sequencing. However, in contrast to the droplet sequencing methods which were available at the time of our study, we were able to first isolate and purify CL and NC monocyte subsets *a priori*, instead of defining cell populations afterward using transcriptional patterns to infer lineages. This method also allowed us to intentionally increase the number of NC monocytes examined, which are comparatively rare in circulation and thus less deeply studied by droplet RNA-seq technology. While RNA-seq would provide total transcriptome data, PCR data is typically more robust and more quantitative, and we found that our quantitative analyses both confirmed and extended the findings observed in the expressed/not expressed analyses. Working with this more limited panel of carefully quantitated genes led to novel insights in our study.

Additional studies will be important to validate our results. We are following up on the on/off pattern of key transcript(s) in another cohort of biologic- and JAK inhibitor-naïve patients. Mechanistic studies in cell culture will allow us to further interrogate these pathways. Synovial tissue studies will allow us to further understand differences among treatment response groups and, where possible, correlate results with findings in the peripheral blood. Further understanding of differences between these patient groups should ultimately allow us to identify alternative targets to exploit therapeutically in patients who would be unlikely to respond to TNFi.

## Data Availability Statement

All the data generated for this study are presented as summary figures in the article/Supplementary Material. Investigators interested in the raw data should contact the authors.

## Ethics Statement

The studies involving human participants were reviewed and approved by Mayo Clinic IRB. The patients/participants provided their written informed consent to participate in this study.

## Author Contributions

TW,WF, ZJ, and TN contributed to the conception and design of the study. TW,WF, ZJ, MJ, JDo, YG-P, BD, DV, KW, TM, SP, CM, JDa, and EM contributed to the acquisition of data. TW, WF, and TN contributed substantially to the analysis and interpretation of data. WF wrote the first draft of the manuscript. TW and TN wrote subsequent drafts. All authors contributed to manuscript revision, read, and approved the submitted version.

## Conflict of Interest

TN holds research grants from EMD Serono and Microdrop, Inc. which are not related to this study. TN and TW have filed a patent on biomarker testing for the prediction of drug response in rheumatoid arthritis. TW served on an Advisory Board for Novartis and as a consultant for Regeneron, unrelated to this study. JDa has received grant funding from Pfizer and has participated on advisory boards for Abbvie and Sanofi, unrelated to this study. The remaining authors declare that the research was conducted in the absence of any commercial or financial relationships that could be construed as a potential conflict of interest.
