# Supplementary material for: Distinct Single Cell Gene Expression in Peripheral Blood Monocytes Correlates With Tumor Necrosis Factor Inhibitor Treatment Response Groups Defined by Type I Interferon in Rheumatoid Arthritis
Source: Front Immunol. 2020 Jul 16;11:1384. doi: 10.3389/fimmu.2020.01384 (PMC7378891; doi:10.3389/fimmu.2020.01384)
Supplement: Supplementary file 1 [file Table_1.docx]

**Supplemental Table 1.** Transcripts examined by qPCR in single monocytes from RA patients.

| **Transcript** | | | | | | | |
| --- | --- | --- | --- | --- | --- | --- | --- |
| *ARG1* | *CD11c* | *CXCL9* | *IDO1* | *IL12* | *IRAK1* | *PRDM1* | *TLR4* |
| *BDCA1* | *CD127* | *CXCR3* | *IFI27* | *IL15* | *IRAK4* | *RELA* | *TLR5* |
| *BDCA3* | *CD14* | *CXCR5* | *IFIH1* | *IL1A* | *IRF1* | *STAT1* | *TLR7* |
| *C1QC* | *CD16* | *CXCR7* | *IFIT1* | *IL1B* | *IRF8* | *STAT2* | *TLR8* |
| *CCL22* | *CD32a* | *E2-2* | *IFIT2* | *IL2* | *JAK1* | *STAT3* | *TLR9* |
| *CCR2* | *CD32b* | *FCER1G* | *IFIT3* | *IL23* | *MAVS* | *STAT4* | *TNFA* |
| *CCR4* | *CD36* | *FLT3* | *IFIT5* | *IL4* | *MYD88* | *STAT5* | *TRAF6* |
| *CCR5* | *CD64* | *GICL3* | *IFN B* | *IL5* | *NFKB1* | *STAT6-2* | *TRIF* |
| *CCR6* | *CD80* | *GMCSF* | *IFN G* | *IL6* | *OAS1* | *TGFB* | *TYK2* |
| *CD103* | *CD86* | *HLADRB1* | *IFNA 2* | *IL8* | *PDL1* | *TLR2* | *VERSICAN* |
| *CD11b* | *CTLA4* | *ICOS* | *IFNAR1* | *ILT7* | *PKR* | *TLR3* |  |
