# Supplementary material for: Distinct Single Cell Gene Expression in Peripheral Blood Monocytes Correlates With Tumor Necrosis Factor Inhibitor Treatment Response Groups Defined by Type I Interferon in Rheumatoid Arthritis
Source: Front Immunol. 2020 Jul 16;11:1384. doi: 10.3389/fimmu.2020.01384 (PMC7378891; doi:10.3389/fimmu.2020.01384)
Supplement: Supplementary file 2 [file Table_2.docx]

**Supplemental Table 2.** Number of cells isolated.

|  | **Classical** | **Non-classical** | **Total Monocytes** |
| --- | --- | --- | --- |
| Total Isolated | 345 | 423 | 768 |
| Dead | 3 | 64 | 67 |
| Alive | 342 | 359 | 701 |
