# Supplementary material for: Distinct Single Cell Gene Expression in Peripheral Blood Monocytes Correlates With Tumor Necrosis Factor Inhibitor Treatment Response Groups Defined by Type I Interferon in Rheumatoid Arthritis
Source: Front Immunol. 2020 Jul 16;11:1384. doi: 10.3389/fimmu.2020.01384 (PMC7378891; doi:10.3389/fimmu.2020.01384)
Supplement: Supplementary file 3 [file Table_3.docx]

**Supplemental Table 3.** Number of single monocytes in each Type I IFN group.

| **Type I IFN Group** | **Classical** | **Non-classical** | **Total Monocytes** |
| --- | --- | --- | --- |
| IFNβ/α Activity > 1.3 | 92 | 63 | 155 |
| IFNβ/α Activity ≤ 1.3 | 246 | 142 | 388 |
| Type I IFN Activity  Undetected  (T1IFN ND) | 4 | 154 | 158 |
