# Supplementary material for: Distinct Single Cell Gene Expression in Peripheral Blood Monocytes Correlates With Tumor Necrosis Factor Inhibitor Treatment Response Groups Defined by Type I Interferon in Rheumatoid Arthritis
Source: Front Immunol. 2020 Jul 16;11:1384. doi: 10.3389/fimmu.2020.01384 (PMC7378891; doi:10.3389/fimmu.2020.01384)
Supplement: Supplementary file 4 [file Table_4.docx]

**Supplemental Table 4. Retained transcripts for prediction of patient group by multivariate logistic regression.**

| Mo | Transcript | Coefficient | P value |
| --- | --- | --- | --- |
| CL | *JAK1* | -0.35391 | <0.0001 |
| CL | *TLR2* | -0.50455 | 0.0003 |
| CL | *IRF8* | -0.64969 | 0.002 |
| CL | *CD16* | -0.44492 | 0.029 |
| CL | *IL1A* | -0.28175 | 0.035 |
| NC | *CD86* | 0.22951 | 0.001 |
| NC | *HLADRB1* | 0.097105 | 0.016 |
| NC | *IL8* | 0.11685 | 0.002 |
| NC | *PDL1* | 0.099174 | 0.037 |
| NC | *TGFB* | 0.07669 | 0.042 |
| NC | *FCER1G* | -0.25984 | 0.034 |

Mo = monocyte. CL = Classical. NC = Non-classical
