# Supplementary material for: Distinct Single Cell Gene Expression in Peripheral Blood Monocytes Correlates With Tumor Necrosis Factor Inhibitor Treatment Response Groups Defined by Type I Interferon in Rheumatoid Arthritis
Source: Front Immunol. 2020 Jul 16;11:1384. doi: 10.3389/fimmu.2020.01384 (PMC7378891; doi:10.3389/fimmu.2020.01384)
Supplement: Supplementary file 5 [file Image_1.pdf]

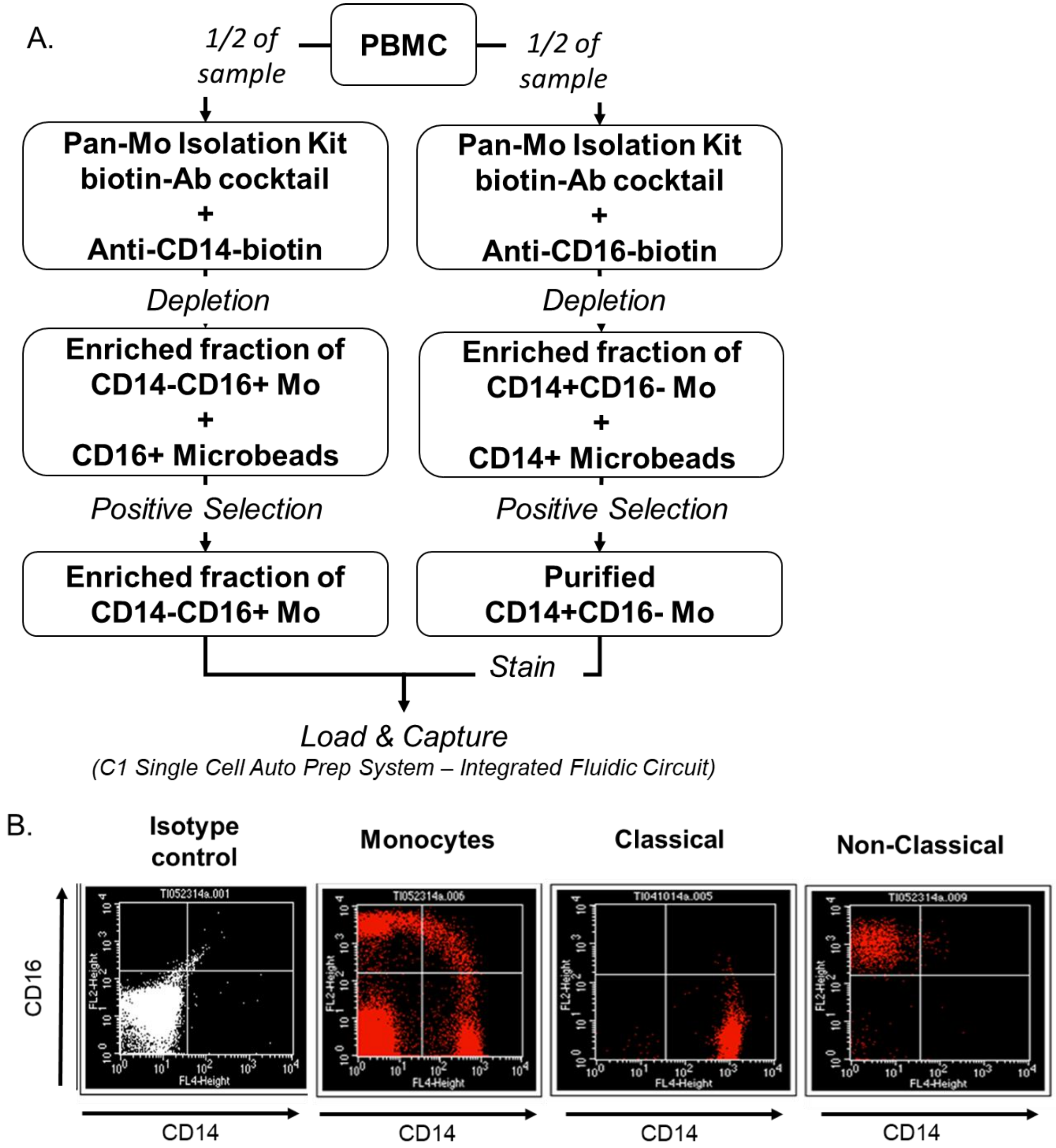

Supplemental Figure 1. Workflow for purification of classical ($CD14^{++}CD16^{-}$) and non-classical ($CD14^{dim}CD16^{+}$) monocytes.

(A). Classical (CL) and non-classical (NC) monocytes (Mo) were isolated from peripheral blood. CL (CD14++CD16−) Mo were purified using the Human Pan-Monocyte Isolation Kit (Miltenyi) with modification of adding anti-CD16-biotin (Miltenyi) into the biotin–antibody cocktail. CD14+ selection (Miltenyi) was used subsequently to further increase purity. Purified CL Mo were stained with Molecular Probes CellTracker Green CMFDA Dye (Life Technologies). NC ($CD14^{dim}$CD16+) Mo were purified similarly, using CD16 microbeads (Miltenyi) during positive selection. (B). Purity checked by flow cytometry was very high (> 95%) for both CL and NC Mo.
