# Supplementary material for: Distinct Single Cell Gene Expression in Peripheral Blood Monocytes Correlates With Tumor Necrosis Factor Inhibitor Treatment Response Groups Defined by Type I Interferon in Rheumatoid Arthritis
Source: Front Immunol. 2020 Jul 16;11:1384. doi: 10.3389/fimmu.2020.01384 (PMC7378891; doi:10.3389/fimmu.2020.01384)
Supplement: Supplementary file 7 [file Image_3.pdf]

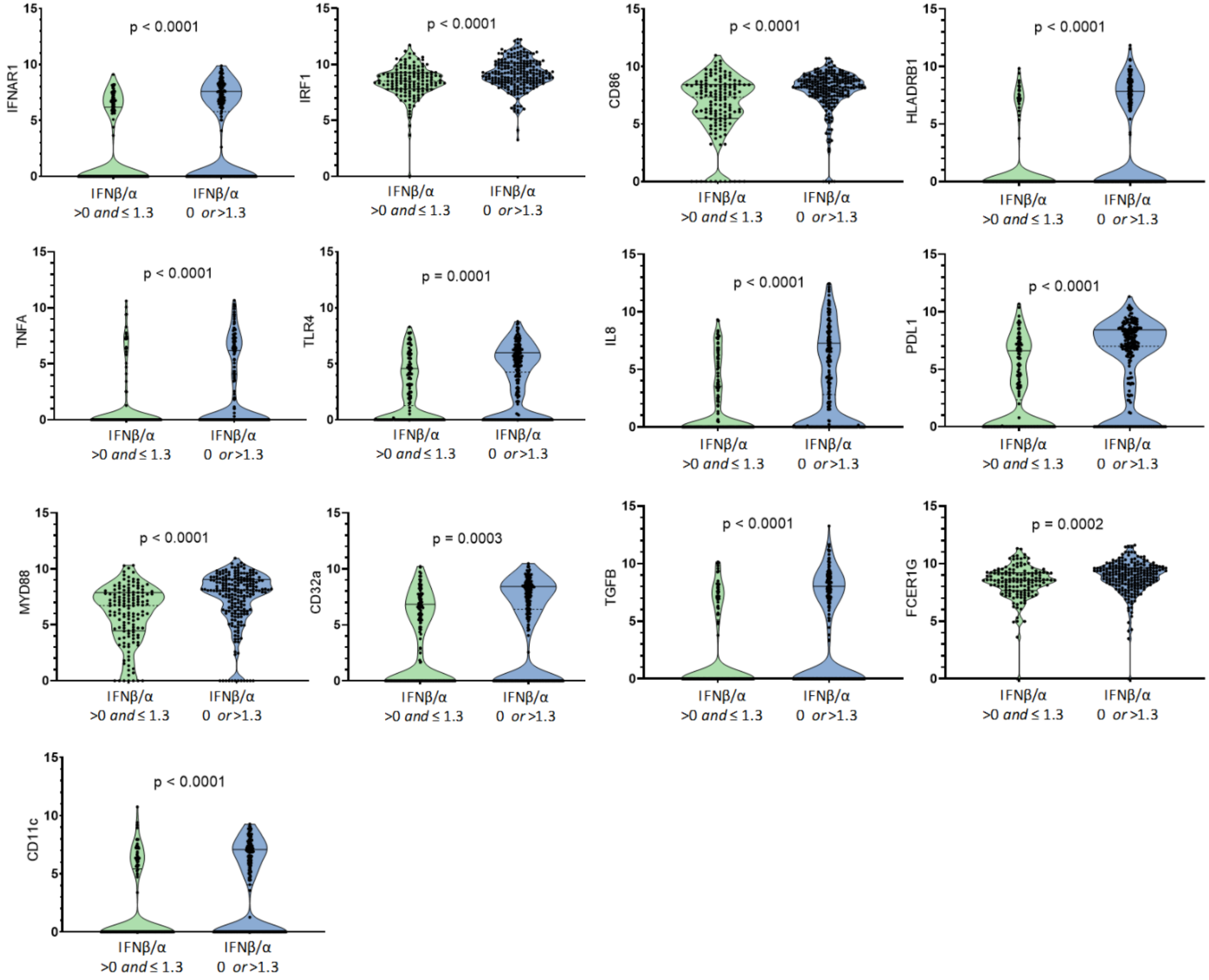

Supplemental Figure 3. Differences in non-classical monocyte expression of genes between IFNβ/α groups when those with undetectable Type I IFN activity are grouped with the IFNβ/α > 1.3 group. T1IFN detectable but IFNβ/α ≤1.3 = IFNβ/α>0 and ≤1.3. T1IFN ND or IFNβ/α >1.3 = IFNβ/α 0 or >1.3. P value determined by Mann Whitney U.
