# Supplementary material for: Distinct Single Cell Gene Expression in Peripheral Blood Monocytes Correlates With Tumor Necrosis Factor Inhibitor Treatment Response Groups Defined by Type I Interferon in Rheumatoid Arthritis
Source: Front Immunol. 2020 Jul 16;11:1384. doi: 10.3389/fimmu.2020.01384 (PMC7378891; doi:10.3389/fimmu.2020.01384)
Supplement: Supplementary file 8 [file Image_4.pdf]

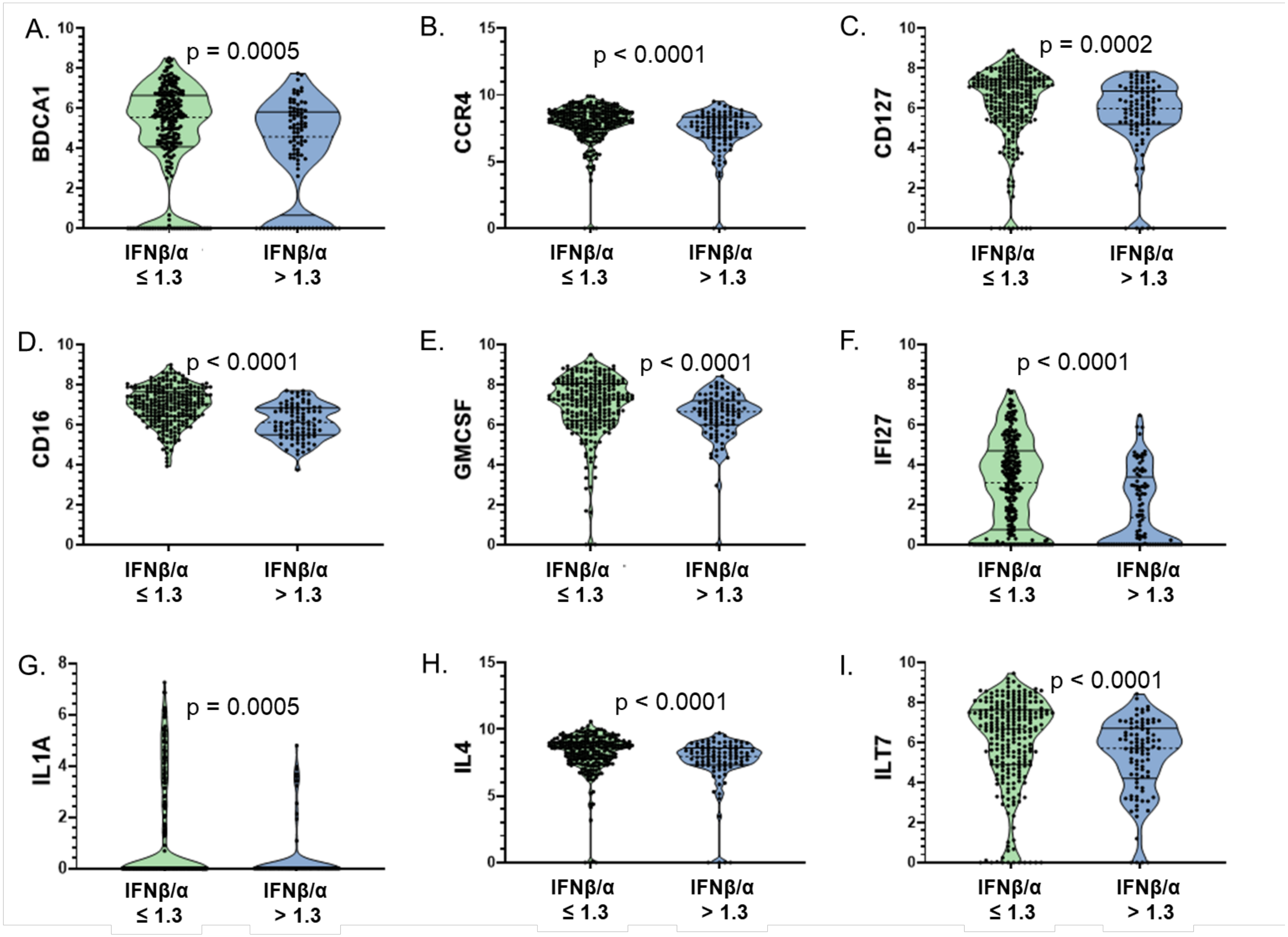

A.
BDCA1
p = 0.0005
B.
CCR4
p < 0.0001
C.
CD127
p = 0.0002
D.
CD16
p < 0.0001
E.
GMCSF
p < 0.0001
F.
IFI27
p < 0.0001
G.
IL1A
p = 0.0005
H.
IL4
p < 0.0001
I.
ILT7
p < 0.0001
IFNβ/α ≤ 1.3
IFNβ/α > 1.3

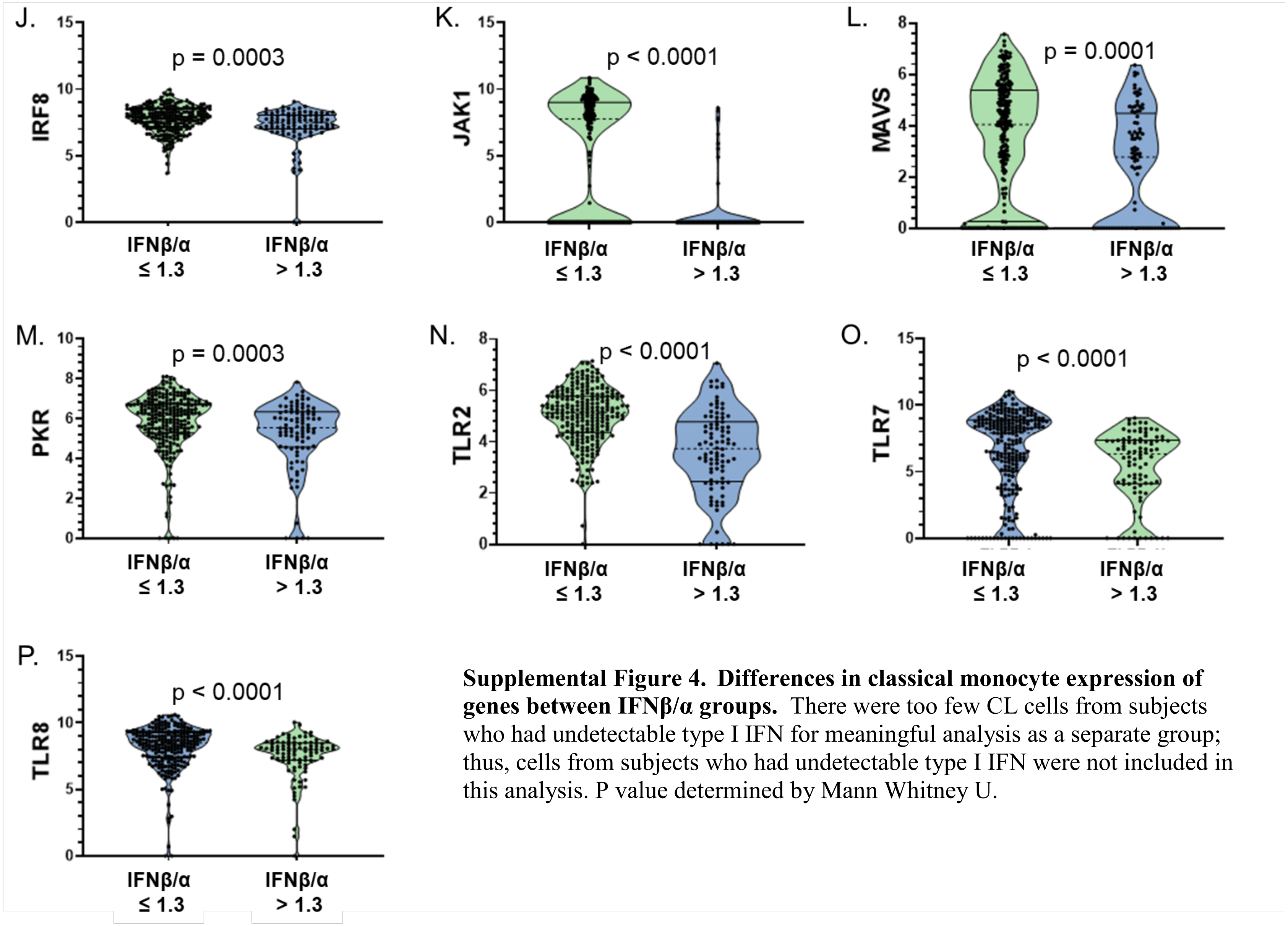

Supplemental Figure 4. Differences in classical monocyte expression of genes between IFNβ/α groups. There were too few CL cells from subjects who had undetectable type I IFN for meaningful analysis as a separate group; thus, cells from subjects who had undetectable type I IFN were not included in this analysis. P value determined by Mann Whitney U.
