# Supplementary material for: Distinct Single Cell Gene Expression in Peripheral Blood Monocytes Correlates With Tumor Necrosis Factor Inhibitor Treatment Response Groups Defined by Type I Interferon in Rheumatoid Arthritis
Source: Front Immunol. 2020 Jul 16;11:1384. doi: 10.3389/fimmu.2020.01384 (PMC7378891; doi:10.3389/fimmu.2020.01384)
Supplement: Supplementary file 9 [file Image_5.pdf]

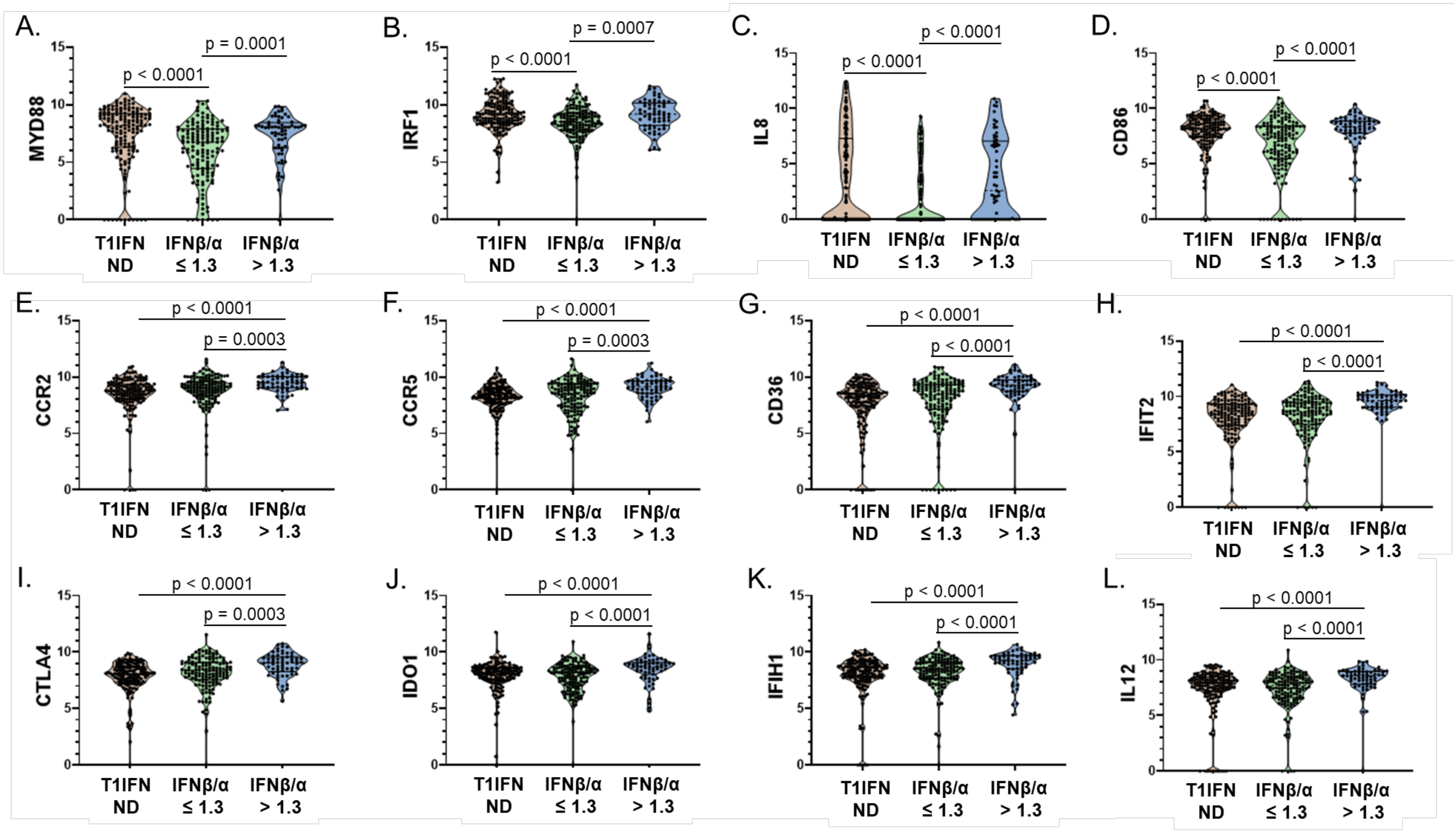
A.
MYD88
p < 0.0001
p = 0.0001
B.
IRF1
p < 0.0001
p = 0.0007
C.
IL8
p < 0.0001
p < 0.0001
D.
CD86
p < 0.0001
p < 0.0001
E.
CCR2
p < 0.0001
p = 0.0003
F.
CCR5
p < 0.0001
p = 0.0003
G.
CD36
p < 0.0001
p < 0.0001
H.
IFIT2
p < 0.0001
p < 0.0001
I.
CTLA4
p < 0.0001
p = 0.0003
J.
IDO1
p < 0.0001
p < 0.0001
K.
IFIH1
p < 0.0001
p < 0.0001
L.
IL12
p < 0.0001
p < 0.0001
T1IFN ND
IFNβ/α ≤ 1.3
IFNβ/α > 1.3

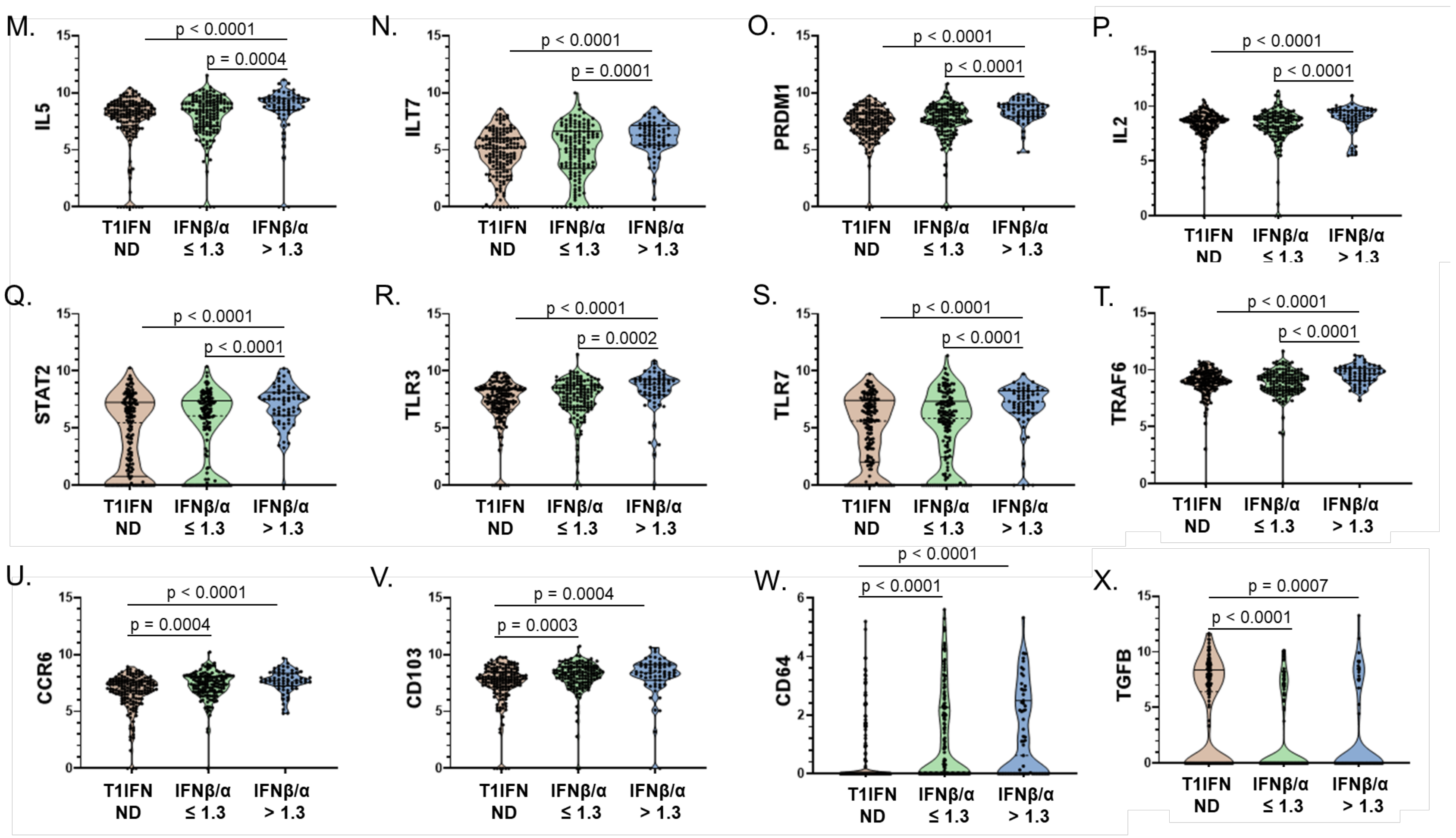

M.
IL5
p < 0.0001
p = 0.0004
N.
ILT7
p < 0.0001
p = 0.0001
O.
PRDM1
p < 0.0001
p < 0.0001
P.
IL2
p < 0.0001
p < 0.0001
Q.
STAT2
p < 0.0001
p < 0.0001
R.
TLR3
p < 0.0001
p = 0.0002
S.
TLR7
p < 0.0001
p < 0.0001
T.
TRAF6
p < 0.0001
p < 0.0001
U.
CCR6
p < 0.0001
p = 0.0004
V.
CD103
p = 0.0004
p = 0.0003
W.
CD64
p < 0.0001
p < 0.0001
X.
TGFB
p = 0.0007
p < 0.0001
T1IFN ND
IFNβ/α ≤ 1.3
IFNβ/α > 1.3

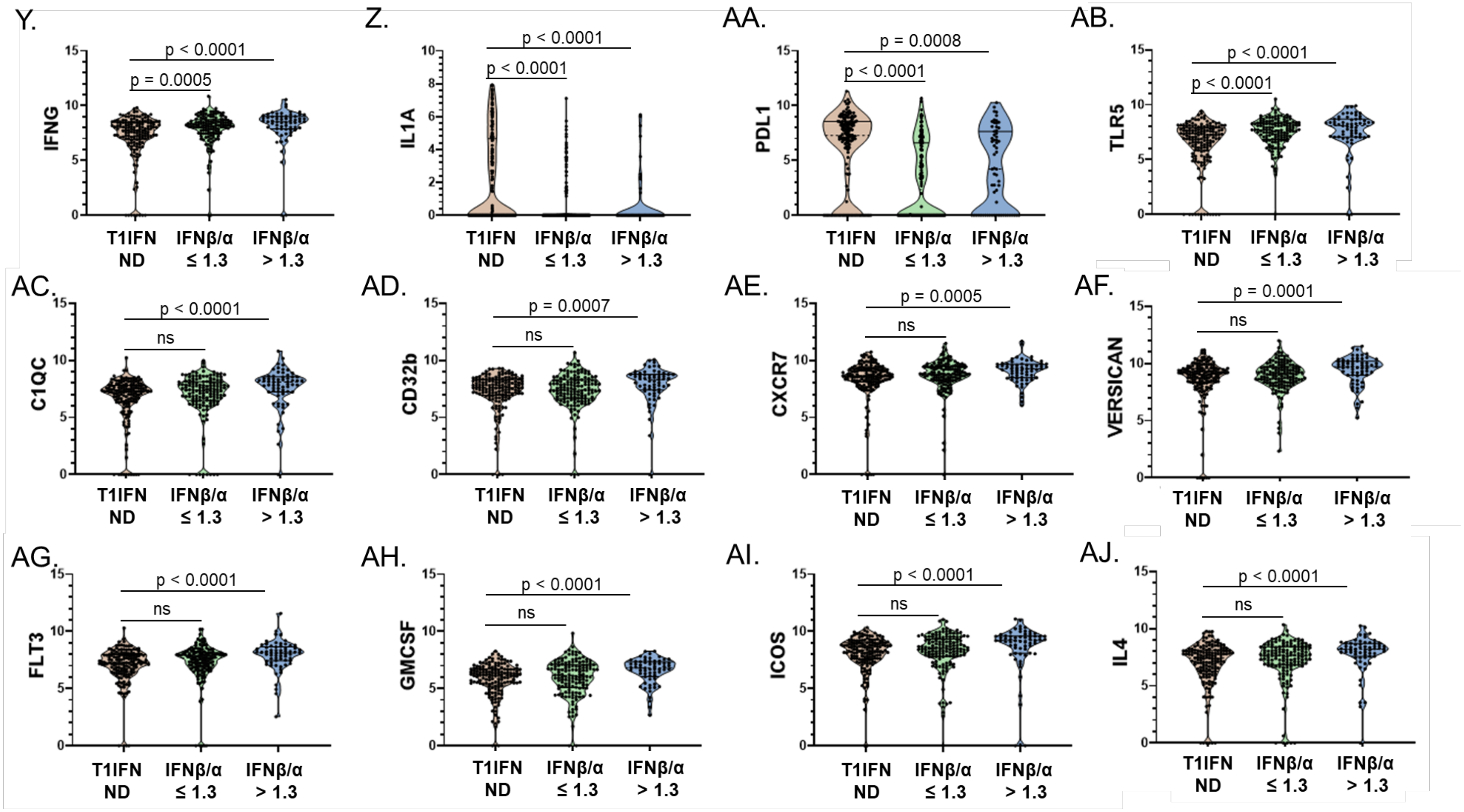

Y.
p < 0.0001
p = 0.0005
IFNG
Z.
p < 0.0001
p < 0.0001
IL1A
AA.
p = 0.0008
p < 0.0001
PDL1
AB.
p < 0.0001
p < 0.0001
TLR5
AC.
p < 0.0001
ns
C1QC
AD.
p = 0.0007
ns
CD32b
AE.
p = 0.0005
ns
CXCR7
AF.
p = 0.0001
ns
VERSICAN
AG.
p < 0.0001
ns
FLT3
AH.
p < 0.0001
ns
GMCSF
AI.
p < 0.0001
ns
ICOS
AJ.
p < 0.0001
ns
IL4
T1IFN ND
IFNβ/α ≤ 1.3
IFNβ/α > 1.3

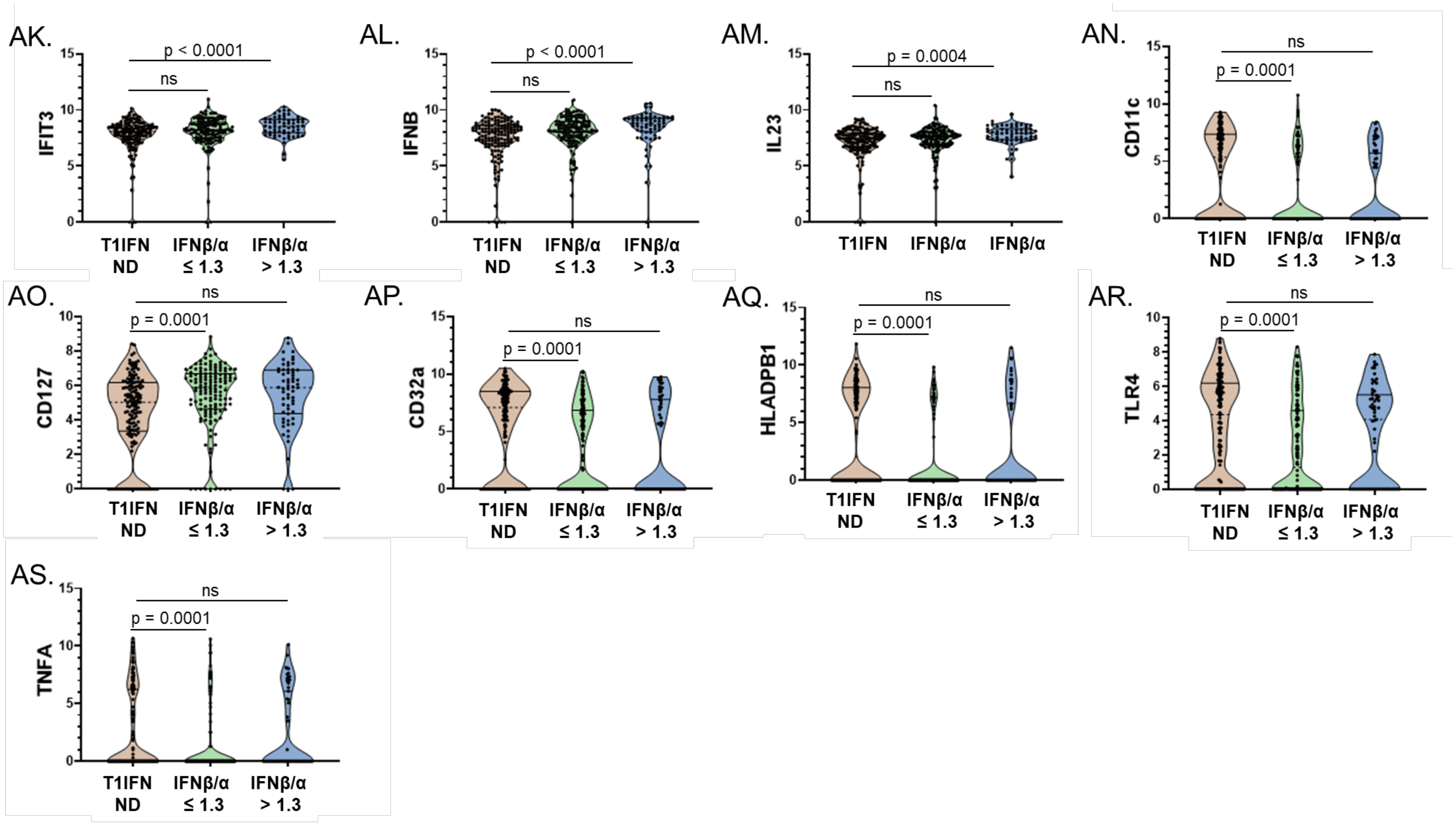

Supplemental Figure 5. Differences in single non-classical monocyte expression of genes between Type I IFN activity groups. P value determined by Mann Whitney U. For (U – AS), difference between IFNβ/α > 1.3 and IFNβ/α ≤ 1.3 was not detected (p ≥ 0.0008).
