# Supplementary material for: Distinct Single Cell Gene Expression in Peripheral Blood Monocytes Correlates With Tumor Necrosis Factor Inhibitor Treatment Response Groups Defined by Type I Interferon in Rheumatoid Arthritis
Source: Front Immunol. 2020 Jul 16;11:1384. doi: 10.3389/fimmu.2020.01384 (PMC7378891; doi:10.3389/fimmu.2020.01384)
Supplement: Supplementary file 10 [file Image_6.pdf]

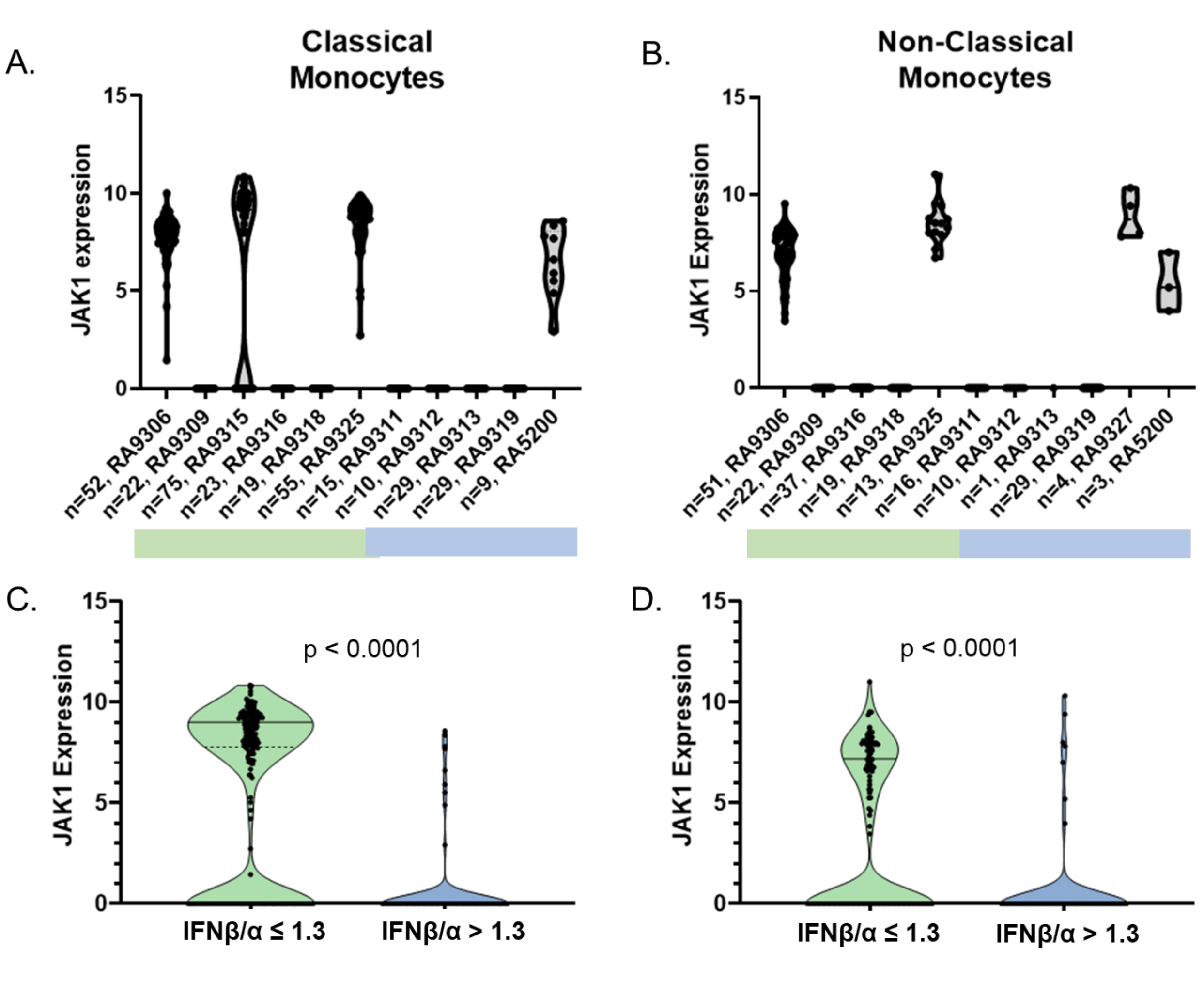

Supplemental Figure 6. Expression of JAK1 in single classical and non-classical monocytes from RA patients who had detectable type I IFN activity. P-value by non-parametric Mann Whitney U. Top panels show each individual patient's cells in a separate column, bottom panels show cells from all patients in aggregate.
