# Supplementary material for: Distinct Single Cell Gene Expression in Peripheral Blood Monocytes Correlates With Tumor Necrosis Factor Inhibitor Treatment Response Groups Defined by Type I Interferon in Rheumatoid Arthritis
Source: Front Immunol. 2020 Jul 16;11:1384. doi: 10.3389/fimmu.2020.01384 (PMC7378891; doi:10.3389/fimmu.2020.01384)
Supplement: Supplementary file 11 [file Image_7.pdf]

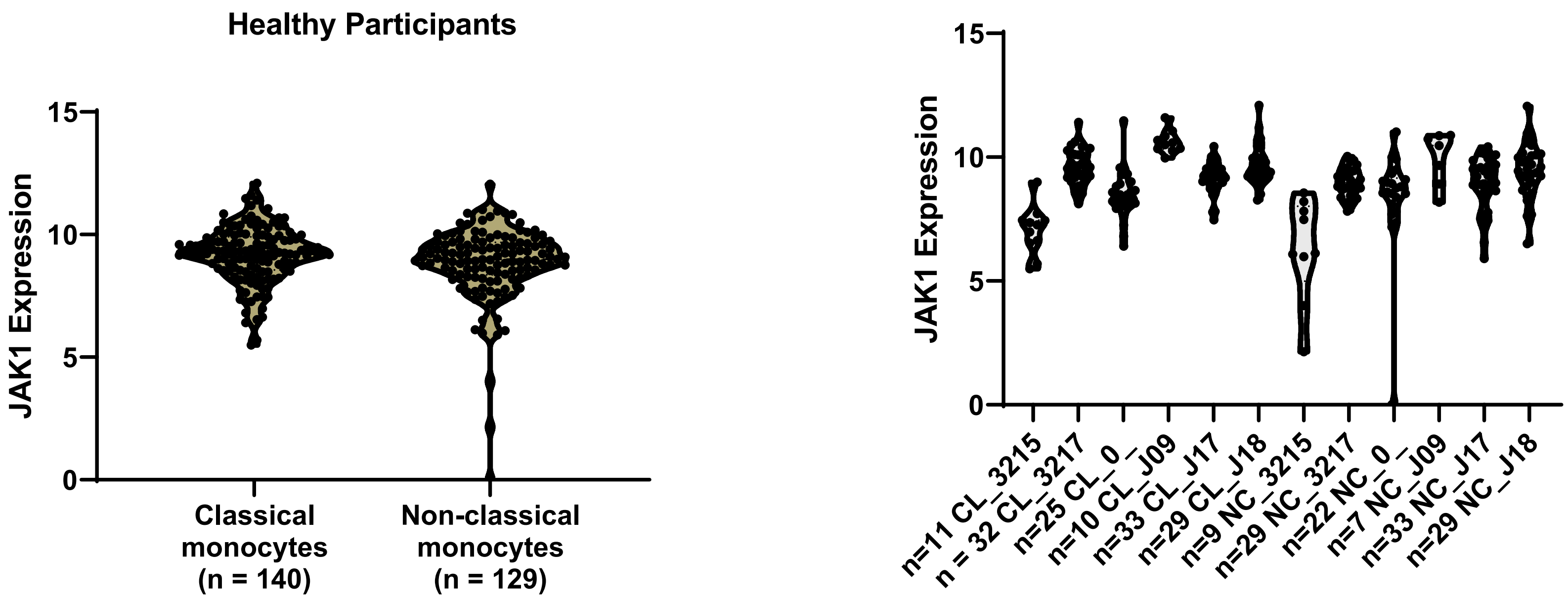

Supplemental Figure 7. Expression of JAK1 in single classical and non-classical monocytes from healthy controls. Left panel shows cells from healthy control in aggregate. The right panel shows each individual healthy participant's cells in a separate column.
