# Supplementary material for: Distinct Single Cell Gene Expression in Peripheral Blood Monocytes Correlates With Tumor Necrosis Factor Inhibitor Treatment Response Groups Defined by Type I Interferon in Rheumatoid Arthritis
Source: Front Immunol. 2020 Jul 16;11:1384. doi: 10.3389/fimmu.2020.01384 (PMC7378891; doi:10.3389/fimmu.2020.01384)
Supplement: Supplementary file 12 [file Image_8.pdf]

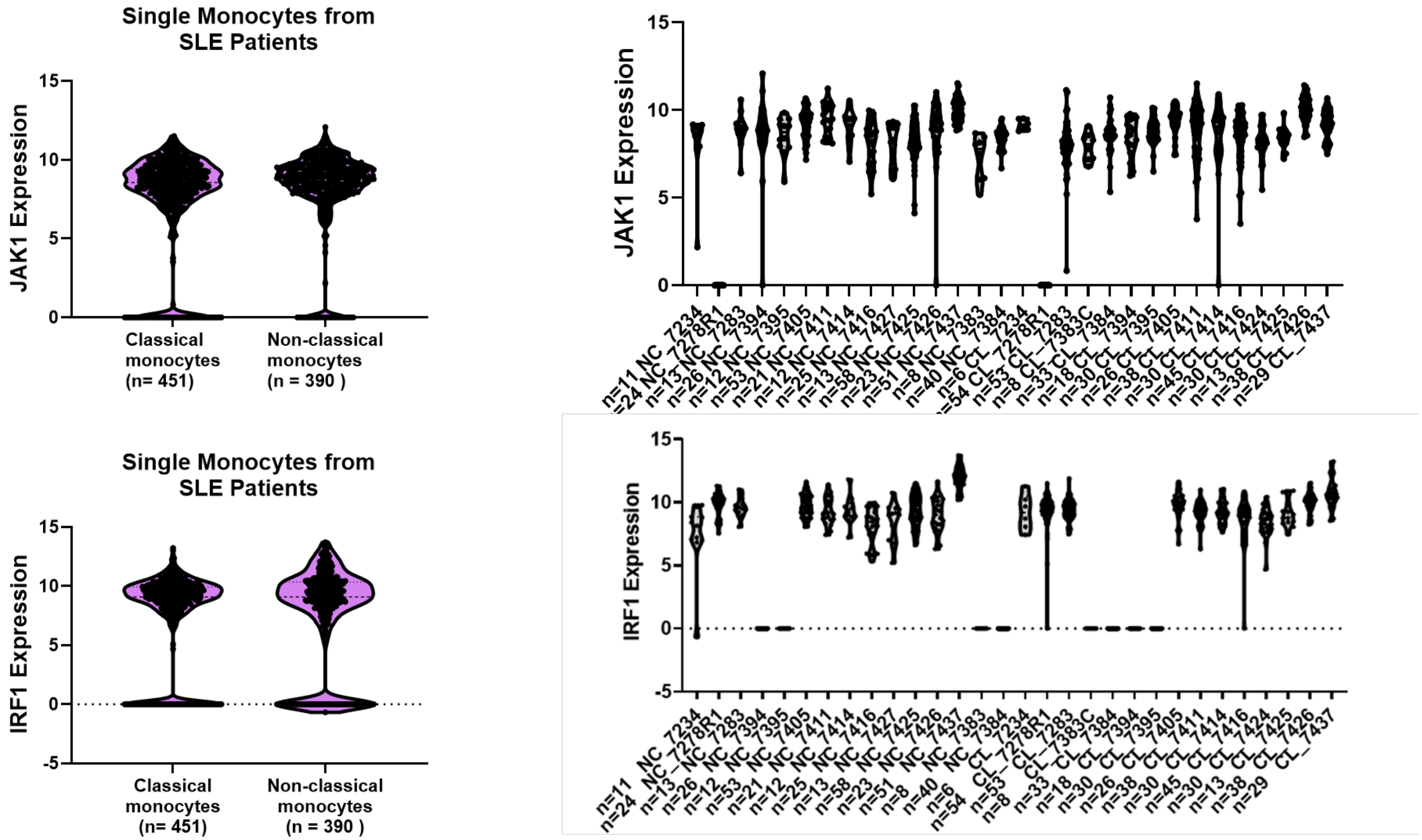

Supplemental Figure 8. Expression of *JAK1* and *IRF1* in single classical and non-classical monocytes from patients with Systemic Lupus Erythematosus. Left panels show cells from all patients in aggregate. Right panels show each individual patient's cells in a separate column.
