# Supplementary material for: Distinct Single Cell Gene Expression in Peripheral Blood Monocytes Correlates With Tumor Necrosis Factor Inhibitor Treatment Response Groups Defined by Type I Interferon in Rheumatoid Arthritis
Source: Front Immunol. 2020 Jul 16;11:1384. doi: 10.3389/fimmu.2020.01384 (PMC7378891; doi:10.3389/fimmu.2020.01384)
Supplement: Supplementary file 13 [file Image_9.pdf]

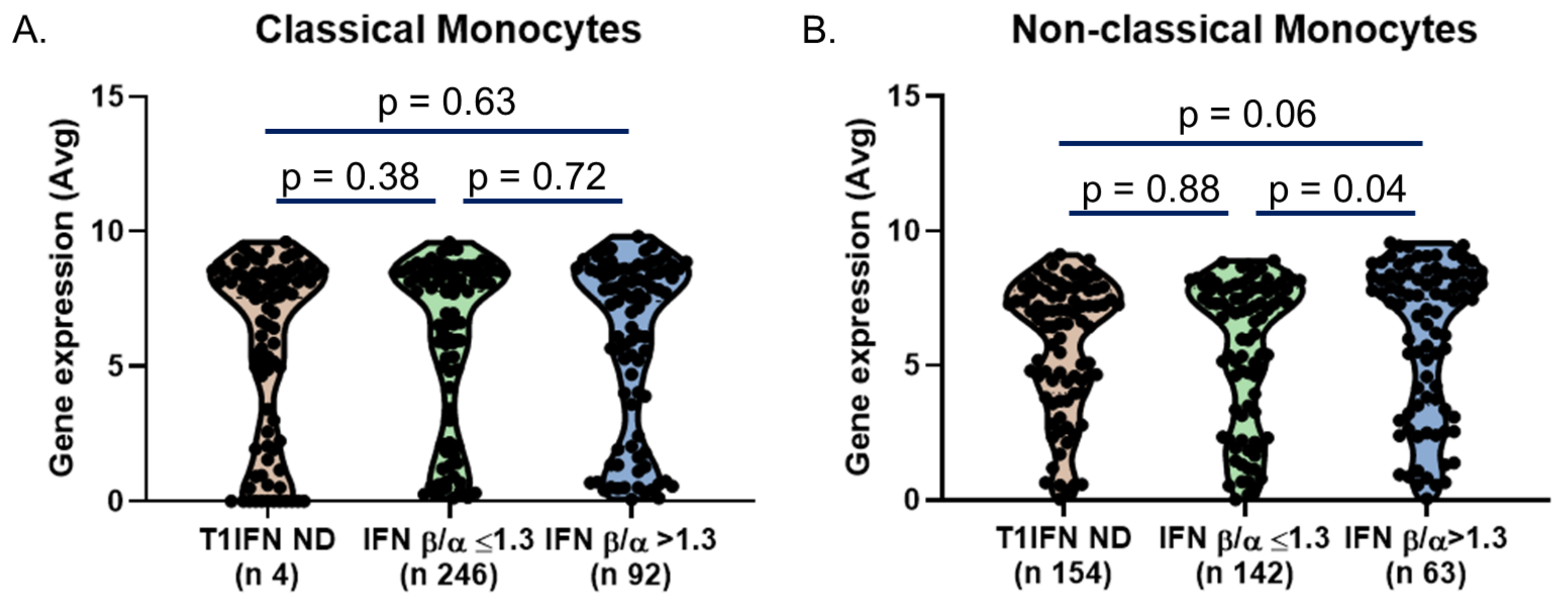

Supplemental Figure 9. Average gene expression in single monocytes by type I IFN group. (A). Classical monocytes. (B). Non-classical monocytes. Each dot represents a single transcript. Number of cells contributing to each group is indicated below the group name (x-axis).
