# Supplementary material for: Distinct Single Cell Gene Expression in Peripheral Blood Monocytes Correlates With Tumor Necrosis Factor Inhibitor Treatment Response Groups Defined by Type I Interferon in Rheumatoid Arthritis
Source: Front Immunol. 2020 Jul 16;11:1384. doi: 10.3389/fimmu.2020.01384 (PMC7378891; doi:10.3389/fimmu.2020.01384)
Supplement: Supplementary file 14 [file Image_10.pdf]

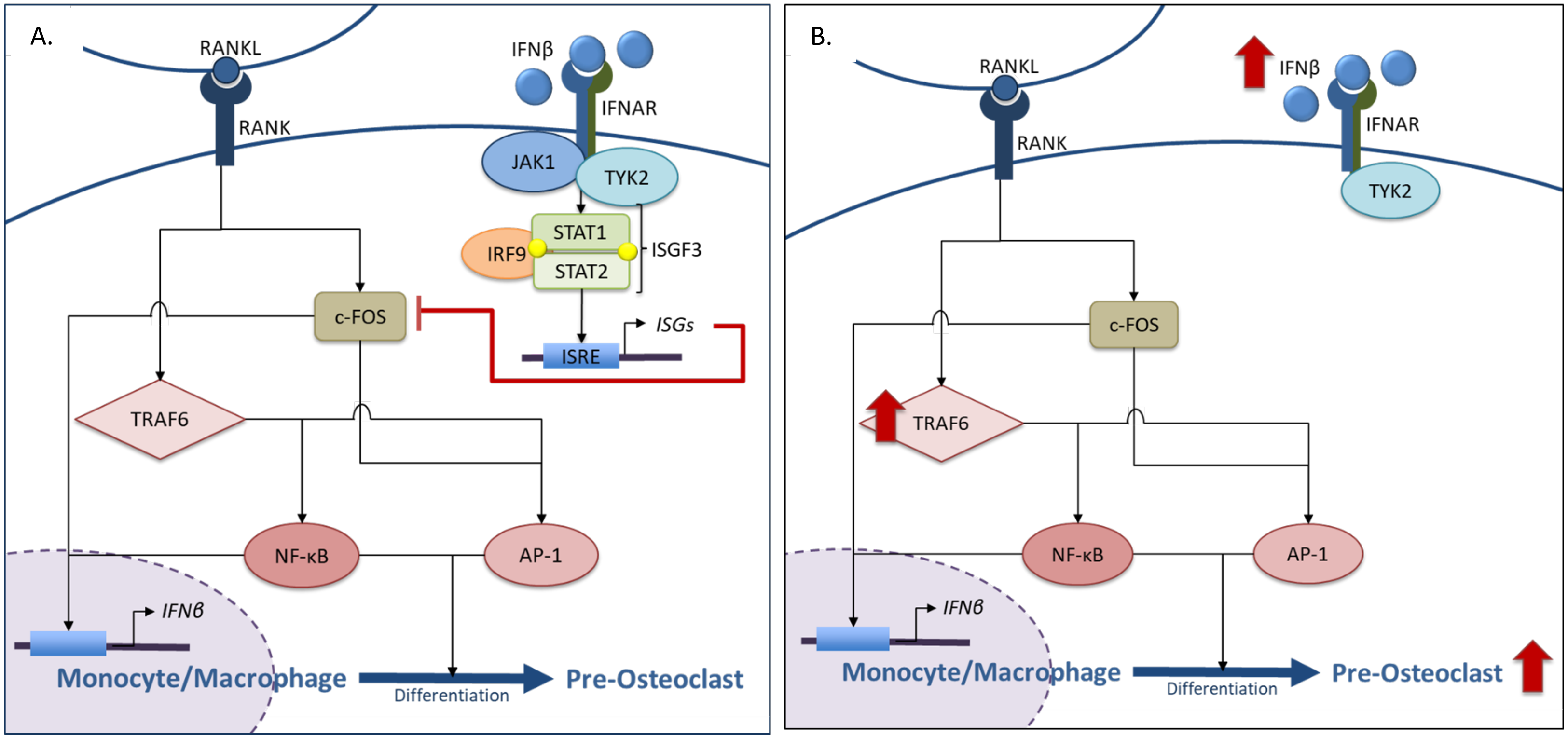

Supplemental Figure 10. IFNβ, ISGs, and osteoclastogenesis. IFNβ production is stimulated as a result of signaling through tumor necrosis factor receptors (TNFRs), of which RANK is an example. Interactions of RANK with RANK ligand (RANKL) activates pathways that promote production of IFNβ and osteoclastogenesis. (A). IFNβ promotes transcription of genes that inhibit osteoclastogenesis. (B). In absence of JAK1, type I IFNs cannot induce the canonical type I IFN pathway, which includes genes that inhibit osteoclastogenesis.
