# Supplementary figures and images for: Distinct Single Cell Gene Expression in Peripheral Blood Monocytes Correlates With Tumor Necrosis Factor Inhibitor Treatment Response Groups Defined by Type I Interferon in Rheumatoid Arthritis
Source: Front Immunol. 2020 Jul 16;11:1384. doi: 10.3389/fimmu.2020.01384 (PMC7378891; doi:10.3389/fimmu.2020.01384)

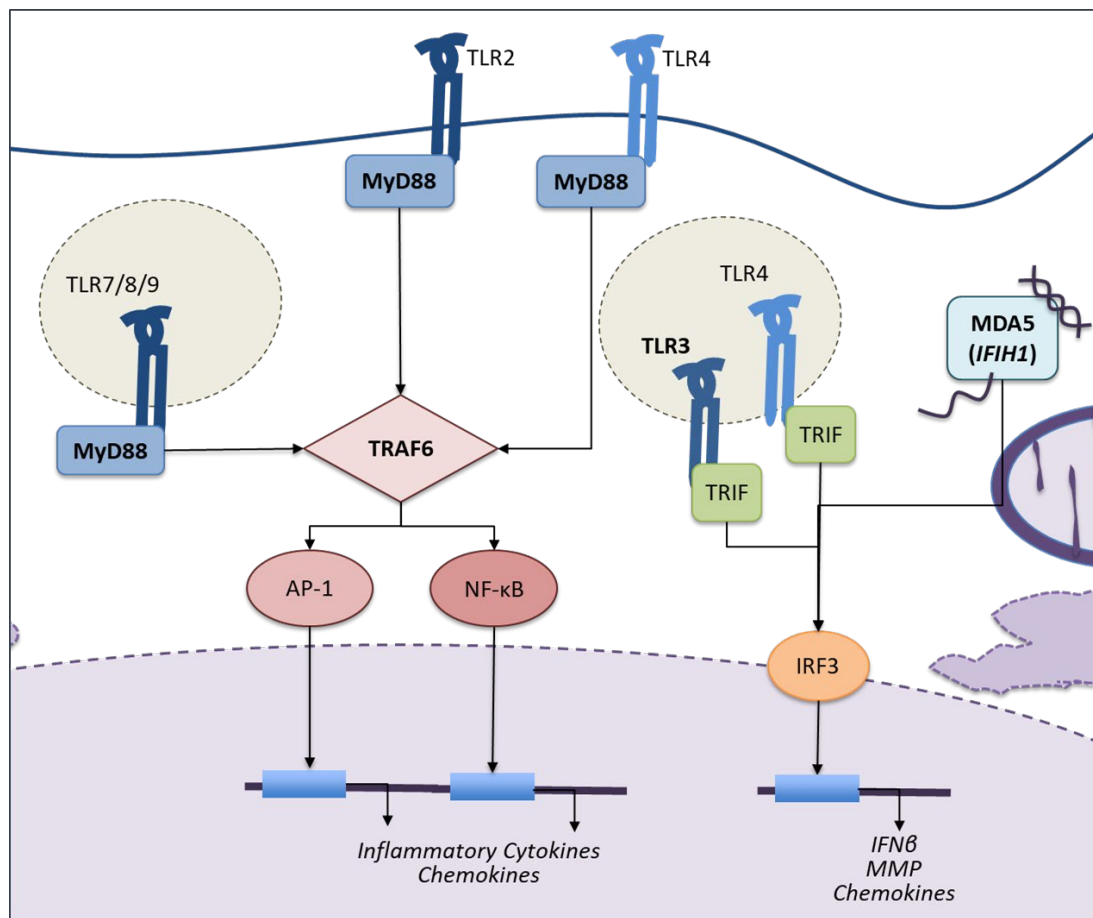

**Supplemental Figure 11. MyD88-TRAF6, TLR3, and MDA5 pathways in monocytes.**

Supplement: Supplementary file 15 [file Image_11.pdf]
